# Novel insights in cryptic diversity of snow and glacier ice algae communities combining 18S rRNA gene and ITS2 amplicon sequencing

**DOI:** 10.1093/femsec/fiad134

**Published:** 2023-10-25

**Authors:** Daniel Remias, Lenka Procházková, Linda Nedbalová, Liane G Benning, Stefanie Lutz

**Affiliations:** Paris Lodron University of Salzburg, Department of Ecology and Biodiversity, Hellbrunnerstr. 34, 5020 Salzburg, Austria; University of Applied Sciences Upper Austria, Stelzhamerstr. 23, 4600 Wels, Austria; Charles University, Faculty of Science, Department of Ecology, Viničná 7, 128 44 Praha, Czech Republic; Charles University, Faculty of Science, Department of Ecology, Viničná 7, 128 44 Praha, Czech Republic; German Research Centre for Geoscience, GFZ, 14473 Potsdam, Germany; Department of Earth Sciences, Freie Universität Berlin, 12249 Berlin, Germany; German Research Centre for Geoscience, GFZ, 14473 Potsdam, Germany

**Keywords:** cryosphere, eDNA, glacier ice algae, ITS2 secondary structure, next generation sequencing, snow algae, species delimitation

## Abstract

Melting snow and glacier surfaces host microalgal blooms in polar and mountainous regions. The aim of this study was to determine the dominant taxa at the species level in the European Arctic and the Alps. A standardized protocol for amplicon metabarcoding using the 18S rRNA gene and ITS2 markers was developed. This is important because previous biodiversity studies have been hampered by the dominance of closely related algal taxa in snow and ice. Due to the limited resolution of partial 18S rRNA Illumina sequences, the hypervariable ITS2 region was used to further discriminate between the genotypes. Our results show that red snow was caused by the cosmopolitan *Sanguina nivaloides* (Chlamydomonadales, Chlorophyta) and two as of yet undescribed *Sanguina* species. Arctic orange snow was dominated by *S. aurantia*, which was not found in the Alps. On glaciers, at least three *Ancylonema* species (Zygnematales, Streptophyta) dominated. Golden-brown blooms consisted of *Hydrurus* spp. (Hydrurales, Stramenophiles) and these were mainly an Arctic phenomenon. For chrysophytes, only the 18S rRNA gene but not ITS2 sequences were amplified, showcasing how delicate the selection of eukaryotic ‘universal’ primers for community studies is and that primer specificity will affect diversity results dramatically. We propose our approach as a ‘best practice’.

## Teaser

Communities of psychrophilic microalgae causing blooms in melting snow and on glaciers in Europe and the European Arctic were evaluated using optimized molecular primers.

## Introduction

The phenomenon of snow and glacier surface discolorations caused by microalgal blooms during the melting season has long been known. On a global scale, this cryoflora is recognized to play an important role in primary productivity in otherwise hostile cryogenic environments (Williamson et al. 2019, Hoham and Remias 2020). Moreover, coloured snow and ice cause accelerated melting due to biological albedo reduction, which in turn affects sea level rise (Hotaling et al. 2021). Yet, there is no comprehensive overview regarding the biodiversity of such extremophilic phototrophs at the species level, nor is there a standardized methodological protocol. Therefore, we present here a best practice approach, with an optimized workflow including consistent sampling, light microscopy-based morphological guidance, the generation of appropriate reference sequences and a final manual verification of taxonomic assignments. The protocol was tested on 18 snow and ice samples from the Alps and the European Arctic.

Classical studies of cryoflora (mainly based on light microscopy) have provided limited information due to poor morphological differentiation of the predominant, often uniform algal cell types. In most cases, these are dominated by either red spherical algal cysts (in snow) or dark purple, short algal filaments (on ice). This led to early assumptions that few cosmopolitan species of green algae dominate worldwide (Kol 1968). At the end of the 20th century, Sanger sequencing using molecular markers improved this knowledge, and overturned older concepts of low phototrophic biodiversity on snow and ice of glaciers and ice sheets. In the last decade, the advent of high-throughput sequencing (HTS; Oliveira et al. 2018), has enabled community characterization at the species level, making a molecular approach feasible for various habitats and questions, and allowing characterizations of the entire biocenosis, including bacteria, archaea, fungi, and protozoa, which are invariably associated with pigmented snow and glacier ice algal blooms.

The first HTS study targeting snow-dominated cryo-habitats was carried out by Lutz et al. (2015a) in samples from Iceland, using a section of the 18S rRNA gene marker for eukaryotes. They found that green alga of the genus *Chloromonas* were the most dominant in snow. Using the same marker in samples from Svalbard, Lutz et al. (2015b) revealed differences in community composition between Arctic green and red snow, which were also reflected in differences in snow chemistry and metabolic profiles. The first biogeographic HTS survey for these habitats compared red snow algal samples from Greenland, Svalbard, Iceland, and northern Sweden (Lutz et al. 2016). The communities were found to be largely uniform with respect to the most abundant taxa, regardless of different location-specific geochemical and mineralogical factors. Furthermore, Lutz et al. (2017) compared distinct habitats in the Arctic (green/red snow, biofilm, ice, cryoconite holes), and demonstrated the functional differences between communities of glacier and snow algae by combining amplicon sequencing, targeted metabolomic and physico-chemical analyses. However, most of the studies relied solely on the more general 18S rRNA marker gene, and no taxonomic refinements were made beyond automated species assignments against the SILVA database (extended by an additional 223 sequences of cryophilic algae; Lutz et al. 2016).

Segawa et al. (2018) accomplished the first bipolar comparison of the red snow phenomenon. Using the 18S rRNA gene and ITS2 markers, they concluded that in polar regions, these blooms consist mainly of cosmopolitan '*Sanguina*' phylotypes (ʿ*Chlamydomonasʾ—*snow group B). They also include endemic phylotypes (ʿ*Chlamydomonasʾ—*snow group A), which are distributed either in the Arctic or Antarctica. Davey et al. (2019) used 18S rRNA metabarcoding to compare the taxonomic and metabolic profiles of green and reddish snow algal blooms in maritime Antarctica. They reported that coastal communities were dominated by *Chloromonas* spp. independent of the snow colour. Green snow was more protein-rich, while red snow accumulated more carotenoids and lipids, an observation also reported for green and red snow algae-rich samples from SE Greenland (Lutz et al. 2014). Luo et al. (2020) characterized cryoflora communities on King George Island, Maritime Antarctica, using Illumina sequencing of the hypervariable V4 region of the 18S rRNA gene. In addition to the aforementioned genera, they found communities dominated by other green algae, namely red slush caused by *Chlainomonas* sp. and green snow by an undescribed Trebouxiophyceae. Similarly, Soto et al. (2020) investigated snow blooms on the same island, combining amplicon metabarcoding with photophysiological measurements. They found taxonomic differences between sampling sites depending on the dominant bloom colour (green, red, and particularly yellow-brownish for chrysophytes). Engstrom et al. (2020) and Yakimovich et al. (2020) performed amplicon metabarcoding on snow algae from mountain ranges in British Columbia, Canada. While they found elevation gradients between the dominant genera *Chlainomonas, Sanguina*, and *Chloromonas*, mutualism of bacteria and fungi with algae did not appear to be taxon specific. In contrast, Krug et al. (2020) found specific interkingdom connectivity, and thus, distinct algae-bacteria interactions in snow algal blooms in the Austrian Alps. Using a minimum entropy decomposition approach in ITS2, Brown and Tucker (2020) delineated the fine-scale geographic and genetic population structure of *Sanguina* snow algae. They argued against a cosmopolitan distribution of a single species by finding a distinct regional biogeographic molecular structures. In line with that, Procházková et al. (2019) reported a large number of oligotypes in a global comparison of *Sanguina* ITS2 sequences, suggesting the existence of high (intra-specific) genetic variability of the widespread *S. nivaloides* (formerly addressed as cf. *Chlamydomonas nivalis*).

In contrast, streptophytic green algae of the genus *Ancylonema* are responsible for blooms on melting ice surfaces of glaciers and have only recently been recognized as important players in changing the albedo on glacier and ice sheet surfaces (Yallop et al. 2012, Cook et al. 2020, Williamson et al. 2020, Chevrollier et al. 2023). The first amplicon sequencing study of these glacier ice algae (Lutz et al. 2018) documented their distribution along a 100 km long transect on the western margin of the Greenland Ice Sheet, showing the presence of several *Ancylonema*-related oligotypes, with a site-specific distribution. More recently, Winkel et al. (2022) found *Ancylonema* in communities on Iceland, using similar protocols.

Given the current state of the variability in amplicon sequencing protocols, it is clear that amplicon data have to be evaluated with caution. For example, Xiao et al. (2014) showed for phytoplankton that light microscopy tentatively underestimates biodiversity (i.e. small and less abundant cells are neglected), and HTS can lead to misidentification at the species level (either due to limited database entries and/or insufficient length or genetic variability of the chosen marker sequence). To test the potential of the current next-generation sequencing methods, we showed in Lutz et al. (2019), based on a case study of cryoflora in the Austrian Alps, that HTS outputs need to be thoroughly checked when the organisms are poorly represented in sequence databases. Crucial is the appropriate choice of similarity thresholds to cluster sequences into OTUs (or ASVs), as well as the delineation of species (Lutz et al. 2018).

To our knowledge, the workflow of amplicon metabarcoding of snow and glacier ice microalgae communities still lacks a 'best practice' protocol. To fill this gap and improve species-level assignments, we introduce, besides other optimisations, new ITS2 zygnematophycean-specific primers and show that these finally allow for a thorough taxonomic assessment.

## Methods

### Field sampling and microscopy

A total of 18 samples from different locations in the Austrian and Swiss Alps ('Alpine'), as well as from Svalbard and Greenland ('Arctic') were analysed. The geographical origin, GPS position, elevation, date of harvest and type of cryoflora bloom used for Illumina sequencing are summarized in Table 1. Sites were selected based on logistical availability and representation of the most common bloom types in this geographic region (green, orange, red, and golden-brown snow). Field sampling, sample processing and storage prior to analyses were performed as previously described (Lutz et al. 2019). Briefly, surface snow or ice was transferred to sterile 50 ml plastic tubes and stored frozen at –20°C prior until further processing. Separate sample aliquots were collected in 50 ml tubes for light microscopy. Algae were observed and classified directly in their meltwater by light microscopy using either a Leica 700, a Zeiss Axiovert 200 M or a Nikon Eclipse 80i microscope.

**Table 1.** Sampling sites of snow and glacier ice with sample id, location, region, habitat, date of harvest, geographic position, and elevation in meters above sea level.

Sample ID	location	region	habitat characteristics	harvest	GPS	elevation
Seasonal snowfields						
WP117	Tyrolean Alps, Austria	Al	red snow	16 May 2017	N47 12.454 E11 01.366	2011
WP119	Tyrolean Alps, Austria	Al	green snow, 50 cm below white surface	16 May 2017	N47 13.320 E11 01.552	2256
WP127	Tyrolean Alps, Austria	Al	red snow	30 May 2017	N47 13.796 E11 00.750	2406
WP181	Tyrolean Alps, Austria	Al	red snow	06 June 2017	N47 12.533 E11 02.911	2341
WP199	Tverrdalen, Svalbard	Al	orange snow	03 July 2018	N78 11.777 E15 31.325	293
WP203	Plåtaberget, Svalbard	Ar	golden-brown snow slush	05 July 2018	N78 12.053 E15 27.913	525
WP205	Bjørndalen, Svalbard	Ar	red snow	07 July 2018	N78 09.317 E15 18.759	347
Glacier surfaces						
WP165	Tyrolean Alps, Austria	Al	greyish surface (Ötztal Ferner)	30 August 2017	N46 48.289 E10 58.802	2724
WP166	Tyrolean Alps, Austria	Al	greyish surface (Ötztal Ferner)	30 August 2017	N46 48.280 E10 58.804	2728
WP212	Graubünden, Switzerland	Al	greyish surface (Morteratsch Glacier)	23 August 2018	N46 24.416 E9 57.567	2693
MIT12_23	Greenland	Ar	greyish surface (Mittivakkat glacier)	19 July 2012	N65 41.177 W37 52.543	195
SVA13_8	Brøggerhalvøya, Svalbard	Ar	greyish surface (Vestre Brøggerbreen)	20 July 2013	N78 53.668 E11 50.442	196
SVA13_19	Brøggerhalvøya, Svalbard	Ar	greyish surface (Midtre Lovénbreen)	21 July 2013	N78 53.331 E12 2.738	172
SVA13_50	Brøggerhalvøya, Svalbard	Ar	greyish surface (Austre Lovénbreen)	03 August 2013	N78 52.551 E12 9.003	287
TAR13_13	Kiruna, Sweden	Ar	reddish surface (Storglaciären)	03 July 2013	N67 54.252 E18 34.972	1436
GrSI16_5	Greenland	Ar	greyish surface (Western Ice Cap)	31 July 2016	N67 04.462 W49 21.254	1015
GrIS16_9	Greenland	Ar	greyish surface (Western Ice Cap)	05 August 2018	N67 05.389 W48 30.657	1402
GrIS16_10	Greenland	Ar	greyish surface (Western Ice Cap)	05 August 2018	N67 05.487 W48 53.984	1236

Al, Alpine; Ar, Arctic

### DNA Sanger sequencing

To obtain the first ITS2 reference sequences of glacier ice algae and *Trochiscia*-like red cells, virtually monospecific field populations were collected, identified by light microscopy, and subjected to Sanger sequencing. The following unialgal samples were used: *Ancylonema nordenskioeldii* (sample WP211 collected in 2018 and described in Procházková et al. 2021), *Ancylonema alaskanum* (sample WP167 collected in 2017 and described in Procházková et al. 2021), *Chloromonas polyptera* (sample DRAnt023 collected in 2009, Antarctica and described in Remias et al. 2013a) and *Sanguina* sp. *Trochiscia*-type (sample DR74a collected in 2017 in the Austrian Alps, N47°12.503 E11°01.317 and described here). Since ITS2 of glacier ice algae could not be amplified with existing primers in previous studies, new primers had to be developed during this work.

DNA isolation for high biomass samples (WP167, WP211) was carried out using a DNeasy Plant Mini Kit (Qiagen, Germany), while when <20 mg wet biomass was available (DR74a and DRAnt023), DNA was extracted using the Instagene Matrix Kit (Bio-Rad Laboratories, Hercules, CA, USA). ITS2 was amplified from DNA samples by polymerase chain reaction (PCR) using existing primers and new reverse primers (Table S1). Specifically, primer pairs of ITS5+ITS4 and TW81+AB28 were used to amplify ITS2 from DRAnt023 and DR74a, respectively. To generate a longer DNA fragment covering ITS1 partial+5.8S+ITS2+partial 26S rRNA of WP211 and WP167 the primer pairs of ITS1 + LR3 and Zyg_ITS_F+LR3, respectively, were used. For Illumina sequencing, 'ice primer' pairs were selected targeting ITS2 regions (together with part of the 5.8 S rRNA), tested on unialgal *Ancylonema* material (WP211 and WP167) and finally developed and optimized for streptophytic (zygnematophycean) algae: 5.8SbF2 (CGATGAAGAACGCAGCG) (Mikhailyuk et al. 2008) and the new LSULP (AATTCGGCGGGTGGTCTTG (this study). The amplification reactions and PCR mix for WP167 and WP211 were the same as described in Procházková et al. (2018a). In case of DR74a and DRANT023, amplification reactions were as follows: each 20.52 μL PCR reaction for amplification of 18S rRNA and rbcL genes contained 1 μL of DNA isolates (diluted to concentration of 5 ng μL^–1^), 4.32 μL 5x MyTaq Red Reaction Buffer (Bioline, Meridian Bioscience, USA), 1.08 μL of each 10 μM primer, 13.82 μL sterile Milli–Q water, and 0.22 μL of 5 U μL^–1^ MyTaq HS Red DNA polymerase (Bioline, Meridian Bioscience, USA); amplification reactions were performed using the following cycle parameters: initial denaturation for 3 min at 95°C, followed by 35 cycles (denaturation for 15 s at 95°C, annealing for 30 s at 59°C (18S rRNA gene), extension for 40 s at 72°C), and final extension for 7 min at 72°C. PCR products were purified and sequenced using an Applied Biosystems automated sequencer (ABI 3730xl) at Macrogene Europe (Amsterdam, The Netherlands). The ITS2 sequences obtained were submitted to the NCBI Nucleotide sequence database (accession numbers: *A. nordenskioeldii* WP211- OL898470, *A. alaskanum* WP167–OL898466, *C. polyptera* DRAnt023–OL898471, *Sanguina* sp. DR74a *Trochiscia*-type—OL962698).

### DNA Illumina sequencing

DNA was extracted using the PowerSoil DNA Isolation Kit (MoBio Laboratories). 18S rRNA gene and ITS2 rRNA amplicons were prepared according to the Illumina ‘16S Metagenomic Sequencing Library Preparation’ guide (https://support.illumina.com/content/dam/illumina-support/documents/documentation/chemistry_documentation/16 s/16s-metagenomic-library-prep-guide-15044223-b.pdf) and as previously described (Lutz et al. 2018). In brief, 18S rRNA genes were amplified using the eukaryotic primers 528F (5′ GCGGTAATTCCAGCTCCAA) and 706R (5′ AATCCRAGAATTTCACCTCT; Cheung et al. 2010) spanning the V4–V5 hypervariable regions. For green, orange, red, and golden-brown snow, ITS2 genes were amplified using the 'snow primers' 5.8SbF (5′ GATGAAGAACGCAGCG; one base shorter from Mikhailyuk et al. 2008) and ITS4R (5′ TCCTCCGCTTATTGATATGC; White et al. 1990). For glacier ice algal samples, 'ice primer' pairs targeting ITS2 regions were selected (Table S1). The pooled library was sequenced on the Illumina MiSeq using paired 300 bp reads at the University of Bristol Genomics Facility.

### Bioinformatics analyses

The quality of each amplicon library and potential sequence trimming were evaluated using FastQC. Subsequent processing steps were carried out in qiime2 (v.2019.1.0). The demultiplexed 18S and ITS2 libraries were individually imported into qiime2 (type: ‘SampleData[PairedEndSequencesWithQuality’).

### Processing of 18S rRNA gene sequences

18S paired-end reads were quality filtered, trimmed, and denoised into ASVs using dada2. The first 10 bp of each read were trimmed off. Forward reads were truncated at 250 bp and reverse reads at 200 bp in order to remove low quality regions. ASVs were annotated using a Naive Bayes classifier pre-trained on the full-length Silva (v.132) database. Sequences matching bacterial and archaeal DNA were removed from the 18S dataset. The feature table was rarified using the lowest common number of sequences per sample (i.e. 61 000) and only ASVs with a minimum frequency of 10 across all samples were retained. For a better overview of the algal community composition sequences annotated with 'Chloroplastida' and 'Ochrophyta' were extracted. The 51 most abundant ASVs (ASVs containing >400 sequences across all samples) were manually BLASTed against the NCBI nt database.

### Processing of ITS2 snow sequences

ITS2 regions of forward and reverse reads were extracted separately using itsxpress ('trim-pair-output-unmerged') and subsequently filtered, trimmed, and denoised into ASVs using dada2. Reads were not further trimmed. The Qiime compatible UNITE database of all eukaryotes (v.8.0) was imported into Qiime2 and the reference reads and taxonomy were used to train a Naive Bayes classifier. Reference sequences were annotated using the pre-trained classifier. ASVs with a minimum frequency of 10 across all samples were filtered out. For a better overview of the algal community composition sequences annotated with 'Viridiplantae' and 'Chromista' were extracted and retained in a separate algal feature table. It was also noted that some algal sequences were annotated as 'Protista' or 'Unassigned'. Therefore, sequences with these annotations were extracted and the 51 most abundant ASVs in the snow and glacier ice algae datasets were manually BLASTed against the NCBI nt database. Sequences matching algae were retained and added to the algal feature table. Again, the 51 most abundant ASVs in this table were manually BLASTed against the NCBI nt database. Sequences matching to algae were retained and added, and non-algal sequences were removed until the 44 or 51 most abundant ASVs represented algal sequences in the snow and glacier ice algae datasets, respectively.

### Processing of ITS2 ice sequences and secondary structure

ITS2 ice paired-end reads were quality filtered and denoised into ASVs using dada2. All taxonomic assignments of representative ASVs were performed by blast search against the nucleotide database in NCBI (see the 'ASV assignment and ITS2 secondary structure' section). The methods of annotation and prediction of the secondary structure of the ITS2 regions were the same as in Lutz et al. (2019) and Remias et al. (2020). The secondary structure of nuclear rRNA ITS2 of were drawn using VARNA version 3.9 (Darty et al. 2009). CBC searches were conducted in the entire ITS2 secondary structure (Procházková et al. 2018a, Pröschold and Darienko 2020, Yakimowich et al. 2021).

### ASV assignment and ITS2 secondary structure

For the most abundant ASV IDs in this study, ASV assignment was performed by blast search against NCBI. In the case of 18S rRNA gene sequences, an identity threshold of ∼99.4% had to be passed in order to be considered as a database match (i.e. a maximum of 2 bp nucleotide difference in a 342 bp sequence was allowed; Lutz et al. 2019). Sequences below this threshold were recorded as 'no blast hit'. For ITS2, an identity threshold of ∼89% against a reference was required to be considered as a database match (Lutz et al. 2019). Sequences below this threshold were recorded as 'no blast hit'.

### Haplotype network

The alignments of the 19 most abundant *A. nordenskioeldii, A. alaskanum*, and *Ancylonema* sp. ASVs and the 35 most abundant *Sanguina nivaloides, Sanguina aurantia, Sanguina* sp. DR74a, and *Sanguina* sp. H14 ASVs were used in the consensus secondary structure modelling and haplotype networks. In the case of *Sanguina* sp. H14, the graph was supplemented by the seven most closely related published ITS2 sequences from the states of Colorado and Washington, USA. (KX063717, KX063721, KX063724, KX063726, KX063729, KX063731, and KX063742; Brown et al. 2016). Each of these most abundant ASVs contributed more than by 1% to the ITS2 sequence data in at least one of the samples. A haplotype was delineated at the 100% similarity threshold (i.e. two sequences belong to the same haplotype if they are identical). Haplotype networks were used to demonstrate the intraspecific diversity (within a species) and geographical distribution of each haplotype (Škaloud et al. 2015). The nuclear network for ITS2 was constructed using TCS software version 1.21 (Clement et al. 2000) and statistical parsimony with a connection limit of 95%. The final edit of the haplotype network was done in Inkscape.

## Results

A total of 18 European Arctic and Alpine habitats were investigated to test the improved metabarcoding approach. These showcase blooms included three red snows in the Alps, one red and one orange snow in Svalbard, one reddish bloom on a glacier in Sweden, and finally 10 locations of bare ice surfaces in the Alps, Svalbard, and Greenland. These 16 communities were selected as 'representative' of melting snow and ice ecosystems in this region. Two less common phenomena of facultative snow algae were also included, namely sub-surface green snow (caused by algae atypical for cryoflora) below white snow in the Austrian Alps, and golden-brown snow slush caused by chrysophytes in Svalbard.

### Total eukaryotic community compositions

**Figure 1.**
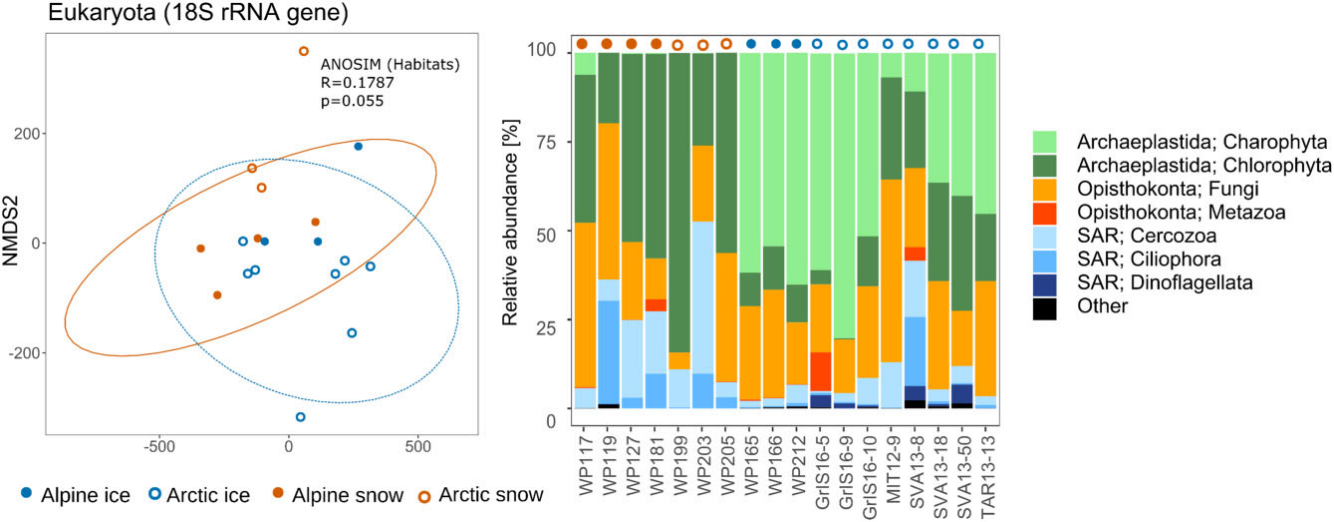
The total eukaryotic community composition and similarities of snow and glacier surface communities based on amplicon metagenomics of the 18S rRNA marker. The samples were assigned to one of four habitat classes and accordingly labelled in the plot and above the bars: 'alpine snow' (orange circle), 'arctic snow' (orange ring), 'alpine ice' (blue circle), and 'arctic ice' (blue ring).

Figure 1 visualizes the abundance of the major eukaryotic taxonomic groups in each sample of melting snow and glacier icer algae habitats, based on metabarcoding of the 18S rRNA gene sequence marker. Microalgae [Chlorophyta, Charophyta (= Streptophyta)] were most abundant, followed by fungi and the SAR group (Cercozoa, Ciliophora, and Dinoflagellata). Samples did not cluster by sites or habitat in the non-metric multidimensional scaling (NMDS) plots. Compared to all other samples, the Arctic golden-brown bloom (WP203) was dominated by chrysophytes only and contained, furthermore, almost no fungi. Results for prokaryote communities can be found in the supplement (Table S2, Fig. S14).

### Snow and glacier ice algal community composition

**Figure 2.**
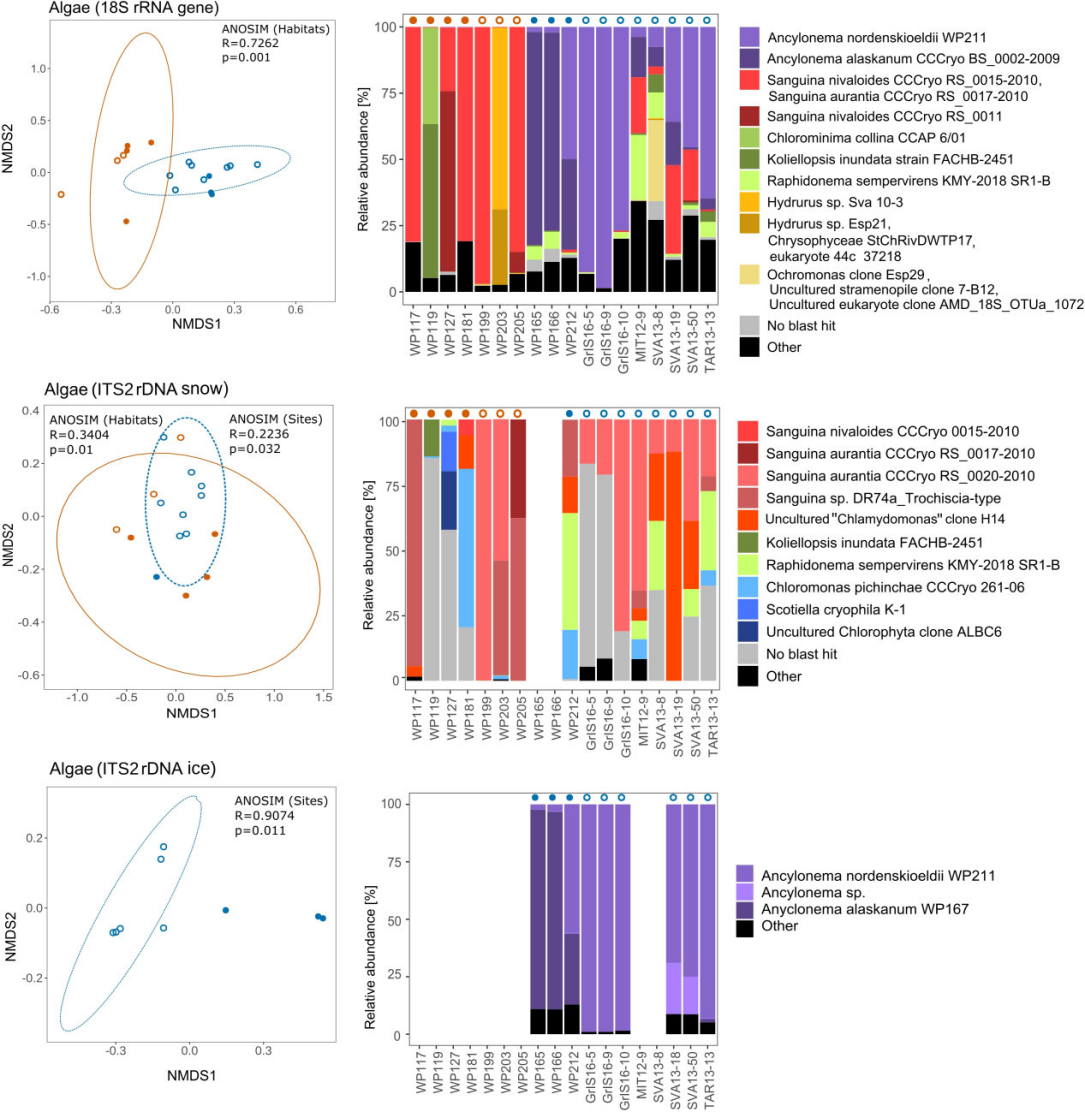
The snow and glacial algal community compositions based on the 18S rRNA and ITS2 markers. Similarities between samples are shown in the NMDS plots and the most abundant taxa are represented in the corresponding bar plots. For visualization, up to 10 most dominant species (ASVs) were selected for each sample. The NMDS plots are shown to the left and the according relative molecular abundances in bar charts to the right. The samples were assigned to one of four habitat classes and accordingly labelled in the plot and above the bars: 'alpine snow' (orange circle), 'arctic snow' (orange ring), 'alpine ice' (blue circle), and 'arctic ice' (blue ring).

The Alpine and Arctic communities were compared using two different primer pairs (18S rRNA gene, ITS2). In the case of ITS2, two different primers ('snow' and 'ice') had to be used to cover more members of the cryoflora. Figure 2 shows the NMDS plots and the corresponding relative read abundances for microalgae in bar charts. In all NMDS plots, samples were assigned to one of four habitat classes: 'alpine snow', 'arctic snow', 'alpine ice', and 'arctic ice'. Analysis of similarity (ANOSIM) for the 18S rRNA gene showed a high overlap between each of the snow and ice habitats (R= 0.7262, *P*=0.001), regardless of whether they were arctic or alpine. Using, ITS2 snow primers, diversity showed no significant difference between Arctic and Alpine sites (R = 0.2236, *P*=0.032) and habitat types (R = 0.3404, *P*=0.01). In the case of the ITS2 ice primer, glacier ice algae habitats differed between Alpine and Arctic locations (R = 0.9074, *P* = 0.011).

In a subsequent step, the 18S rRNA gene and ITS2 marker sequences were used to perform a detailed description of the eukaryotic phototrophs for each site, and to assign the ASVs according to reference databases. The bar charts in Fig. 2 show the most abundant algal ASVs found in the communities. More extensive lists of abundant ASVs for the 18S rRNA gene and for the two ITS2 markers can be found in the Supplementary Tables S3–S5. These are derived from manually optimized taxonomic assignments using blast against the NCBI database. In detail, 18S rRNA metabarcoding showed that several species of *Sanguina* (6 ASVs) were responsible for red blooms in both the European Arctic and Alps, but the limited length of the marker fragment did not species identification. All ice surfaces were inhabited by glacier algae of the genus *Ancylonema*, with 4 ASV belonging to the filamentous *A. nordenskioeldii* and 1 ASV to the unicellular *A. alaskanum* (formerly '*Mesotaenium berggrenii* var. *alaskana*', referred to as *'A. alaskana'* in Procházková et al. 2021). The latter was most abundant in the samples from the Alps, Svalbard, Sweden, and on a valley glacier in Greenland, but had very few reads in the samples from the Greenland ice sheet. On Greenland's Mittivakkat glacier (MIT 12–9), a mixture of snow algae (*Sanguina*), glacier algae, and a psychrophilic ubiquitous alga of cold regions (*Raphidonema sempervirens*) was found. The sub-surface ('atypical') green snow in the Alps (WP119) was dominated by *Chlorominima* sp. and *Koliellopsis inundata* (Chlorophyceae), both of which were not found in any other sample. In detail, ITS2 secondary structure analyses showed that the first alga was closely related to *Chlorominima collina* CCCryo 273–06 (Fig. S1), but it represented an independent species. A *Chlainomonas* species (ASV read 100% identical to OTU008, and 99.7% identical to: *Chloromonas rubroleosa* CC1, *Chlainomonas* sp. RRD1, *Chlainomonas* sp. Bagley_NODE_5578, and *Chlainomonas* sp. 190526Oze2R) was found in all glacier ice samples and in one Svalbard red snow sample (WP205), but only in low abundance (<5.2% algal reads).

Unicellular Chrysophyceae dominated in Arctic golden-brown snow slush (Svalbard, WP203), and 5 ASVs were affiliated with *Hydrurus* sp. (two of which dominated this site). On Vestre Bröggerbreen in the same archipelago (Sva 13–8), *Ochromonas* clone Esp29 was co-dominant with many other microalgae. In the other melting habitats and in Alpine sites, Chrysophyceae were detected only in very low abundances.

**Figure 3.**
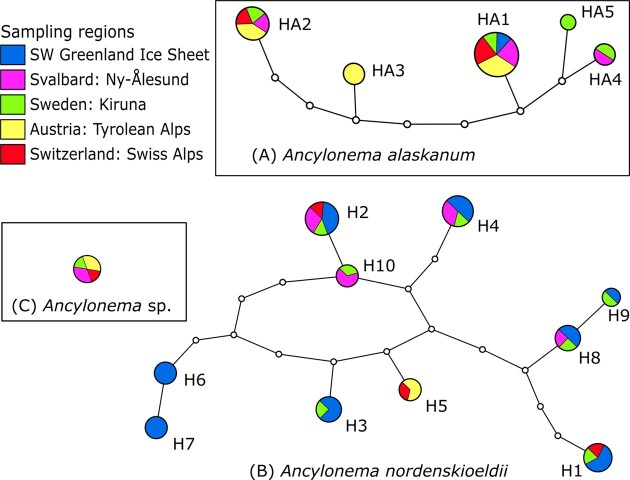
Geographic distribution of glacial algae based on molecular ITS2 haplotypes detected in this study: (A) *A. alaskanum*, (B) *A. nordenskioeldii*, and (C) *Ancylonema* sp. Only the most abundant ASVs were used which accounted for >1% of the ITS2 sequence data in at least one of the samples. Each haplotype network was constructed by a statistical parsimony method with a 95% connection limit. The geographical origin was labelled with colours according to the legend (upper left). Each circle represents a haplotype (i.e. field samples which had identical Illumina ITS2 ASVID). The size of the circle is proportional to the number of sampling sites, which belong to that specific haplotype. Lines connect each haplotype with its most similar relative. Open dots represent mutation steps between haplotypes, one dot indicates a change of one base pair.

**Figure 4.**
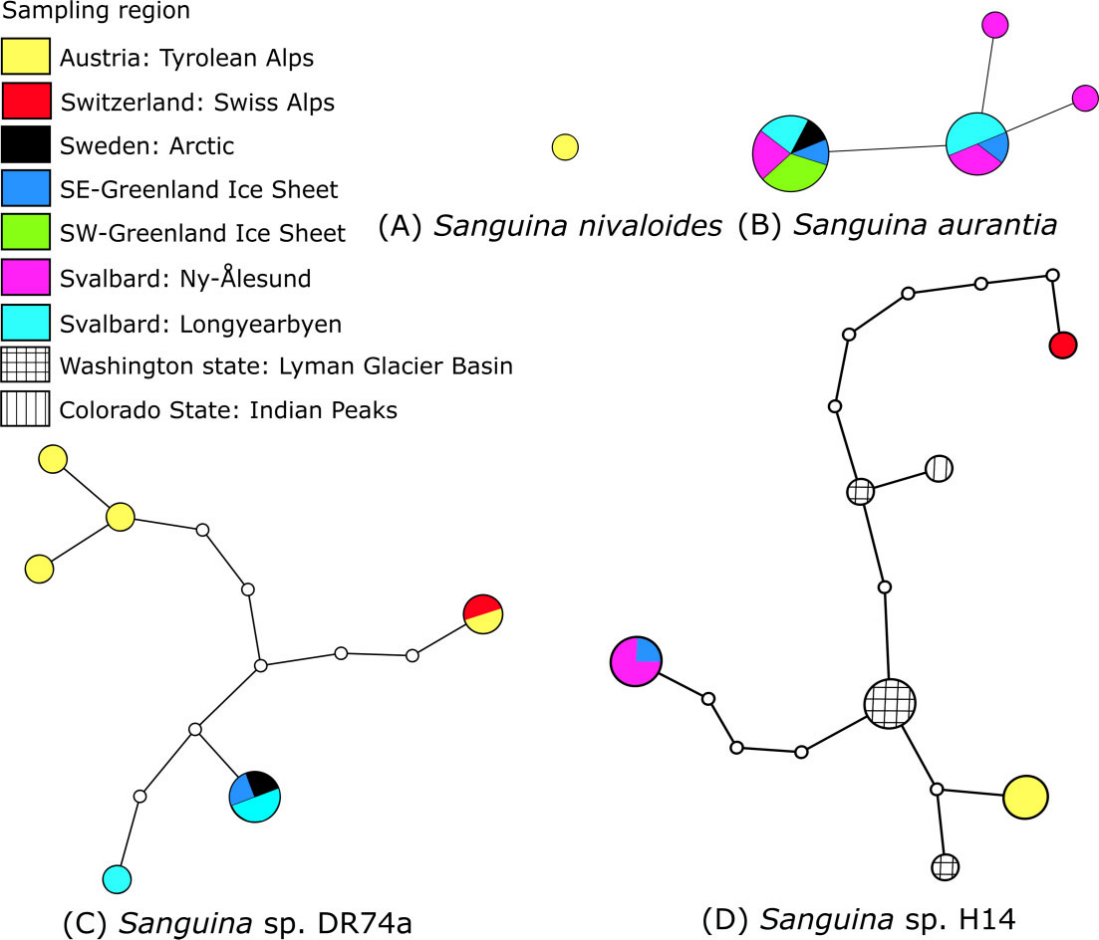
Geographic distribution of *Sanguina* species based on molecular ITS2 haplotypes detected in this study: (A) *S. nivaloides*, (B) *S. aurantia*, (C) *Sanguina* sp. DR74a, and (D) *Sanguina* sp. H14. In case of (D), the graph was supplemented by the seven closest related published ITS2 rDNA sequences, from Colorado and Washington States, U.S.A. (KX063717, KX063721, KX063724, KX063726, KX063729, KX063731, KX063742; Brown et al. 2016). The geographical origin was labelled with colours according to the legend (upper left). Each circle represents a haplotype. The size of the circle is proportional to the number of sampling sites, which belong to that specific haplotype. Lines connect each haplotype with its most similar relative. Open dots represent mutation steps between haplotypes, circle indicates a change of one base pair.

The ITS2 marker amplicons were able to elucidate the diversity in more detail (Figs. S1–13). In most respects, the qualitative taxonomic results were similar to those obtained with the 18S rRNA gene. However, in the case of *Sanguina*, ITS2 accompanied by CBC search in the secondary structure was able to assign ASV sequences at the species level (from the newly generated reference sequence or from the type material BlastN match), including *S. nivaloides* in the Alps (1 ASV), *Sanguina* sp. DR74a in the Arctic and Alps (6 ASVs), for *S. aurantia* only in Arctic habitats (5 ASVs), and for *Sanguina* sp. H14 in the Arctic and Alps (3 ASVs). Based on ITS2 secondary structure comparison (Figs. S2 and S3), the latter also belongs to the genus *Sanguina*, but represents a so far undescribed species (previously referred to as uncultured *'Chlamydomonas'* clone H14 in Brown et al. (2016)). Within the glacier ice algae phototrophic communities distinct geographical patterns were found (Fig. 3): Arctic habitats (in Greenland and Svalbard) were colonized by one to three genetically distinct glacier ice algal species (which was not visible in the 18S rRNA gene sequence data), *A. nordenskioeldii, A. alaskanum*, and *Ancylonema* sp. (Figs. S4–S7, Table S6). The latter undescribed taxon was also found in the Swiss Alps and Sweden, but at much lower abundances (<0.5% of reads) (Table S5). LM suggested that this undescribed species of *Ancylonema* might be filamentous and morphologically distinct from *A. nordenskioeldii*. In contrast, the unicellular *A. alaskanum* was more dominant in Alpine habitats but almost absent from the Greenland Ice Sheet, as indicated by the haplotype network: *A. nordenskioeldii* resulted in ten ITS2 interconnected haplotypes (Fig. 3). The haplotypes differed by one to twelve nucleotide changes out of 194 base pairs. For *A. alaskanum*, five haplotypes were recovered from the dataset, differing from two to eight nucleotide changes out of 197 base pairs. Haplotype networking was also performed for *Sanguina* (Fig. 4). In the case of *Sanguina* sp. DR74a, this resulted in six ITS2 interconnected haplotypes, each with either an Alpine or Arctic distribution. The same pattern was observed for three haplotypes of *Sanguina* sp. H14.

Other members of the glacier algal communities were *R. sempervirens* (Fig. S8), which occasionally contributed up to 45% of ITS2 reads, namely in the Arctic (Svalbard) and the Alps (Switzerland), and an alga related to *Ploeotila* sp. CCCryo086-99 (Fig. S9), abundant at an Arctic site (Greenland Ice Sheet (GrIS)), and *Limnomonas spitsbergensis* CCCryo 217–05 (Fig. S10), observed in the south-eastern GrIS. *Chlainomonas* reads were not recovered in the ITS2 dataset, likely because its amplifiable fragment was too long (about 640 bp including priming sites), and thus preventing it from being sequenced by the sequencing platform used (MiSeq 300 bp PairEnd).

In general, the plausibility of the molecular results regarding the abundances of phototrophs was roughly evaluated and confirmed by light microscopy (data not shown). Four exceptions were found. The first was an Alpine red snow sample (WP181), which showed discrepancies between LM evaluation and ITS2 Illumina abundances: While the molecular data indicated ∼60% *Chloromonas* sp. CCCryo 261–06 (conspecific with *Chloromonas* cf. *alpina* CCCryo 033–99; Fig. S11) and only 18.9% *Sanguina*-like ASVs, the LM showed >90% *Sanguina*-like cysts. In contrast, but less striking in terms of divergent numbers, another Alpine red snow (WP117) contained to some extend *Chloromonas* cells in the LM, but the latter were absent in the ITS2 Illumina data. Thirdly, Alpine red snow WP127 contained a high number of *Sanguina* cysts, but these were absent from the ITS2 molecular results, which were instead dominated by sequences of snow-dwelling *Chloromonas*-like spp. instead, namely *Scotiella cryophila*, Chlorophyta clone ALBC6 (Fig. S12), or an alga related to OTU375 (Fig. S13). Finally, the *Hydrurus*-related Chrysophyceae were generally not amplified with both ITS2 primers used in this study, resulting in a distorted community structure in the corresponding bar chart shown in Fig. 2.

## Discussion

The application of this best practice protocol confirmed that the main members of the local cryoflora are distributed within three genera of green algae: *Chloromonas, Sanguina*, and *Ancylonema* (Hoham and Remias 2020). In the case of the latter two, undescribed species were revealed using ITS2. The likely fourth frequent genus, *Chlainomonas*, which causes red snow, was only marginally covered by this study, as European blooms of this genus are mostly restricted to the melting ice cover of some high-altitude mountain lakes (Procházková et al. 2018b). Since amplicon sequencing can also recover low-abundance taxa, it was possible to detect *Chlainomonas* using 18S rRNA in all glacier ice samples from the European Alps, the Greenland Ice Sheet, and a Swedish glacier. This alga is identical to an alga previously found in Antarctica (Segawa et al. 2018) and very closely related to the records from North America and New Zealand (Novis et al. 2023).

Golden-brown snow caused by unicellular Chrysophyta is closely related to the genus *Hydrurus* (Remias et al. 2013b, Luo et al. 2020, Soto et al. 2020), which was confirmed in this study. Also in the case of chrysophytes, cryoflora communities are dominated by species with very close genetic relationship. Not unexpectedly, this leads to similar cell morphologies and a low variation in certain molecular markers, e.g. 18S rRNA gene or *rbc*L. As a result, HTS studies with snow and glacier ice algae are challenging in terms of revealing true biodiversity or comparing habitats. For example, Nakashima et al. (2021) argued that partial 18S rRNA gene sequences were insufficient for species differentiation within *Sanguina*. In such cases of very close taxa, evaluation of the hypervariable secondary structure of the ITS2 marker is an alternative, as demonstrated here. However, this screening for compensatory base exchanges is currently non-automated and labour-intensive (Segawa et al. 2018, Lutz et al. 2019, Yakimowich et al. 2021).

A key finding of this study was that HTS revealed unexpected biodiversity in at least two cases: first, the improved protocols revealed a third species of glacier ice alga, *Ancylonema* sp. This is consistent with morphological differences: LM indicated that this species is filamentous and can be distinguished from *A. nordenskioeldii* by smaller cell sizes (Procházková et al. 2021). Furthermore, the ITS2 marker showed 91% sequence identity between *A. nordenskioeldii* and *A. alaskanum* (at 100% sequence coverage), and the secondary structures of ITS2 transcripts revealed a single CBC in the middle part of helix I (Fig. S3). This provides good molecular support that both represent two independent species. Secondly, the use of the ITS2 marker was crucial for the species-level elucidation of red snow. The use of metabarcoding combined with ITS2 secondary structure comparison revealed the existence of undescribed species, the two most important being *Sanguina* sp. DR74a and *Sanguina* sp. H14. They differed from the described species *S. nivaloides* and *S. aurantia* in their ITS2 secondary structures. This confirms that the red snow genus *Sanguina* contains more species than currently recognized but their cysts may remain morphologically indistinguishable.

Exceptional results came from two Arctic glaciers (Sva 13–19, Sva 13–50) where both chlorophycean and streptophycean microalgae were co-occurring. This can be explained by natural succession during the melting season: two independent seasonal blooms occurred chronologically. First, chlamydomonadacean flagellates thrive in melting snow, and later, zygnematophycean species grow on the bare ice surface after snow melt (Takeuchi 2013, Lutz et al. 2014). Thus, snow and glacier ice algae can be found together on the same sites during the late melting season, but in our experience there is not necessarily a causal relationship. Such aspects of successional development were not considered in this study due to high logistical demands, hence all sites were sampled only once per season. Moreover, golden-brown communities and green snows can be considered ephemeral, thriving due to either good water or nutrient availability. In such cases, succession can lead to either rapid complete snow melt or transformation into red snows and glacier ice algae blooms on bare ice. The latter ones can be regarded as 'climax stages' until complete snow melt or the onset of the next winter snowfall.

While 'true' cryoflora is thought to reproduce only in snow and on ice (Hoham and Remias 2020), ubiquitous microalgae that can cope well with low temperatures and high irradiance are also found in these communities. This was particularly the case for *Raphidonema* and *Koliella* (Trebouxiophyceae). Under favourable conditions (high availability of liquid water, low irradiance, and high nitrogen input), species atypical of the cryoflora can produce visible blooms, as was the case for Alpine sample WP119. In addition, another dominant trebouxiophycean alga caused green and orange patches in the snow in coastal Maritime Antarctica (Soto et al. 2020, partial 18S rRNA gene: OTU1674, OTU1653, OTU3753). It was initially assigned to 'uncultured *Chlorella*' (accession number AB903015) and is currently assigned to *K. inundata* (accession number MT274431). This is another example of how the quality and constant updating of reference database can be essential for species delimitation.

Snow blooms caused mainly by *Chloromonas* species were not covered by this study, but they are well known from mountainous and coastal polar habitats (Hoham and Remias 2020). However, cells of this genus were present in many of the samples investigated. The newly generated ITS2 reference sequence of *C. polyptera*, obtained from snow adjacent to penguin rockeries in Maritime Antarctica, was used to test the hypothesis of its absence in the northern hemisphere: In fact, there was not a single database match for the Alps and the Arctic. Previous reports of this Antarctic species in HTS studies of the Northern hemisphere (e.g. Lutz et al. 2015a, Terashima et al. 2017) may be due to ambiguous species assignment, as using only a section of the 18S rRNA gene does not distinguish it from other closely related *Chloromonas* species (Lutz et al. 2019).

*Rosetta* cells ('ruby cysts' etc.) were formerly assigned to *Chlamydomonas nivalis* (Kol 1968), their morphology and phylogenetic position is under preparation (Engstrom et Raymond—pers. comm.). In this study, in the three samples (WP181, 127, and WP117), up to 7% of 18S rRNA gene reads were identical to the uncultured algal isolate 0935–5 (accession number LC371440), which morphologically appeared as dark red ellipsoidal cells (based on single-cell Sanger sequencing—see Fig. S22 in Segawa et al. 2018). This finding suggests that some *Rosetta*-like cells can also be found in the European Alps. This is another example of how the understanding of microbial composition is strongly dependent on the quality of reference sequence databases. Indeed, samples WP127 and WP181 showed a high proportion of 'no blast hits' for ITS2 (Fig. 2).

We cannot exclude a technical issue that could be responsible for the observed discrepancies between ITS2 and 18S rRNA gene sequence results in some samples, e.g. sequencing errors that lead to a much higher relative abundance of certain ASVs when compared to the actual abundance of the organism in the sample (due to incorrectly recovered homopolymer segments and carry-forward incomplete-extension errors, Lückling et al. 2014). This can be mitigated by several methodological measures. This includes the generation of OTUs based on phylogenies as monophyletic lineages and the exclusion of very long branches with low abundances that are close to very abundant ASVs, as well as the inclusion of technical replicates, the removal of spurious sequences and unrepresentative OTUs, the use of high stringency clustering methods for out generation, the estimation of treatment effects at higher taxonomic levels, the adaptation of the unique molecular identifier and other newly developed methods to reduce PCR and sequencing errors, and the identification of true low abundance rare species. Combined, these can improve reproducibility (Wen et al. 2017). Nevertheless, Illumina 18S rRNA data, as a proxy for genus-level determination, corresponded well with LM observations for the majority of samples. Therefore, light microscopy guidance is crucial in microalgae community assessment to detect and evaluate possible data inconsistencies. A second issue to generally improve the results is the way the field sampling is done: ideally, harvest into 50 ml tubes should not be done from a single spot, but rather pooled from e.g. five spots of the bloom of 10 ml each within a given square meter to avoid random sampling errors. Overall, a unified method for snow and glacier ice algae sampling, sample preservation, extraction, sequencing and data analyses etc is advised when the aim is to compare data sets across locations.

In our samples, two blooms were caused by facultative cryoflora: subsurface Alpine green snow (WP119), where a dominant alga was shown to be an undescribed taxon, which is closely related to *C. collina*. The latter has been described as a psychrophile from the snow in Maritime Antarctica (Gálvez et al 2021). In addition, we present the another report of *L. spitsbergensis* from Svalbard (Sva13-50), which was originally isolated and previously reported from persistent snowfields at Spitsbergen (Tesson and Pröschold 2022). The widespread occurrence of these algae in snow is not surprising, as they follow different rules than the true cryoflora. They may 'accidentally' bloom in quite water-logged snow or be triggered by external nutrient inputs. In the context of golden-brown snow caused by chrysophytes, more sampling will be needed to elucidate their total biodiversity, and the fact that they were not detected by the ITS2 snow primers leaves their diversity at the species-level unresolved.

In conclusion, the aim of this study was to target the different characteristic cryoflora types of the Alps and the European Arctic in order to assess a deeper level of biodiversity at the species level. In the future, further community metabarcoding using the high-resolution ITS2 marker will help to elucidate which haplotypes (respectively species) dominate under specific conditions. In general, an improved primer pair for ITS2 reading 'glacier ice algae' and 'snow algae' sequences will be mandatory to cover all relevant groups of microalgae in a convenient way. The results of this and similar studies currently point to the presence of both ubiquitous cosmopolites and local species with limited distribution. It remains to be seen whether the latter are either geographically isolated or whether their occurrence depends on unknown ecological conditions.

## Supplementary Material

fiad134_Supplemental_File
