# Correction to: Novel insights in cryptic diversity of snow and glacier ice algae communities combining 18S rRNA gene and ITS2 amplicon sequencing

**DOI:** 10.1093/femsec/fiaf021

**Published:** 2025-06-09

This is a correction to: Daniel Remias et al., Novel insights in cryptic diversity of snow and glacier ice algae communities combining 18S rRNA gene and ITS2 amplicon sequencing, *FEMS Microbiology Ecology*, Volume 99, Issue 12, December 2023, https://doi.org/10.1093/femsec/fiad134.

Supporting data for this research was omitted from the originally published article but is available at https://doi.org/10.5281/zenodo.14726190.

