# Supplementary material for: Novel insights in cryptic diversity of snow and glacier ice algae communities combining 18S rRNA gene and ITS2 amplicon sequencing
Source: FEMS Microbiol Ecol. 2023 Oct 25;99(12):fiad134. doi: 10.1093/femsec/fiad134 (PMC10659120; doi:10.1093/femsec/fiad134)
Supplement: fiad134_Supplemental_File [file fiad134_supplemental_file.docx]

**Novel insights in cryptic diversity of snow and glacier ice algae communities combining 18S rRNA gene and ITS2 amplicon sequencing**

**Daniel Remias^1,2^, Lenka Procházková^3^, Linda Nedbalová^3^, Liane G. Benning^4,5^ and Stefanie Lutz^4,6^**

**^1^** Paris Lodron University of Salzburg, Department of Ecology & Biodiversity, Hellbrunnerstr. 34, 5020 Salzburg, Austria

^2^ University of Applied Sciences Upper Austria, Stelzhamerstr. 23, 4600 Wels, Austria

^3^ Charles University, Faculty of Science, Department of Ecology, Viničná 7, 128 44 Praha, Czech Republic

^4^ German Research Centre for Geoscience, GFZ, Potsdam, 14473 Potsdam, Germany

^5^ Department of Earth Sciences, Freie Universität Berlin, 12249 Berlin, Germany

^6^ Present address: Department of Agroecology and Environment, Agroscope, Zurich, Switzerland

**Corresponding author:** Daniel Remias, E-mail: [daniel.remias@plus.ac.at](mailto:daniel.remias@plus.ac.at)

**Supplementary Table 1:** List of existing and newly developed primers used for amplification of the ITS2 marker (F, forward; R, reverse). “Type” specification is relevant for Illumina sequencing in this study.

| **Primer** | **Type** | **Direction** | **Sequence** | **Reference** |
| --- | --- | --- | --- | --- |
| ITS5 | ITS2 | F | GGAAGTAAAAGTCGTAACAAGG | White et al. (1990) |
| TW81 | ITS2 | F | GGGATCCGTTTCCGTAGGTGAACCTGC | Goff et al. (1994) |
| AB28 | ITS2 | R | GGGATCCATATGCTTAAGTTCAGCGGGT | Goff et al. (1994) |
| ITS1 | ITS2 | F | TCCGTAGGTGAACCTGCGG | White et al. (1990) |
| Zyg_ITS_F | ITS2 | F | TCCGTAGGTGAACCTGCAG | Trumhová (2016) |
| LR3 | ITS2 | R | GGTCCGTGTTTCAAGACGG | Vilgalys and Hester (1990) |
| 5.8SbF | ITS2 snow | F | GATGAAGAACGCAGCG | Mikhailyuk et al. (2008) modified |
| ITS4 | ITS2 snow | R | TCCTCCGCTTATTGATATGC | White et al. (1990) |
| 5.8SbF2 | ITS2 ice | F | CGATGAAGAACGCAGCG | Mikhailyuk et al. (2008) |
| LSULP | ITS2 ice | R | AATTCGGCGGGTGGTCTTG | this study |

| **Supplementary Tables 2**: Bacterial ASVs with a minimum frequency of 100 and their taxonomic identification at the lowest possible level (16S rRNA gene ). | | | | |  |  |  |  |
| --- | --- | --- | --- | --- | --- | --- | --- | --- |
|  |  |  |  |  |  |  |  |  |
| **Affiliation** | **WP117** | **WP119** | **WP127** | **WP181** | **WP205** | **WP165** | **WP166** |  |
| Acidobacteria, Acidobacteriia, Acidobacteriales, Acidobacteriaceae | 0,0 | 0,0 | 0,0 | 0,0 | 0,0 | 0,0 | 0,0 |  |
| Acidobacteria, Acidobacteriia, Acidobacteriales, Acidobacteriaceae | 0,0 | 0,0 | 0,0 | 0,0 | 0,0 | 0,0 | 0,0 |  |
| Acidobacteria, Solibacteres, Solibacterales, Solibacteraceae, Candidatus Solibacter | 0,0 | 0,0 | 0,0 | 0,0 | 0,0 | 0,2 | 0,3 |  |
| Actinobacteria, Acidimicrobiia, Acidimicrobiales | 0,0 | 0,0 | 0,0 | 0,0 | 0,0 | 0,0 | 0,1 |  |
| Actinobacteria, Actinobacteria, Actinomycetales, ACK-M1 | 0,0 | 0,0 | 0,0 | 0,0 | 0,0 | 0,0 | 0,0 |  |
| Actinobacteria, Actinobacteria, Actinomycetales, Intrasporangiaceae | 0,0 | 0,0 | 0,0 | 0,0 | 0,0 | 1,2 | 1,0 |  |
| Actinobacteria, Actinobacteria, Actinomycetales, Microbacteriaceae, Salinibacterium | 0,0 | 0,0 | 0,7 | 0,3 | 0,0 | 4,5 | 3,0 |  |
| Actinobacteria, Actinobacteria, Actinomycetales, Nocardiaceae | 0,0 | 0,0 | 0,0 | 0,0 | 0,0 | 1,6 | 1,3 |  |
| Actinobacteria, Actinobacteria, Actinomycetales, Sporichthyaceae | 0,0 | 0,0 | 0,0 | 0,0 | 0,0 | 1,1 | 0,9 |  |
| Armatimonadetes, Armatimonadia, FW68 | 0,0 | 0,0 | 0,0 | 0,0 | 0,0 | 4,0 | 4,6 |  |
| Bacteroidetes | 16,0 | 0,0 | 34,9 | 20,1 | 0,0 | 8,2 | 7,9 |  |
| Bacteroidetes, Cytophagia, Cytophagales, Cytophagaceae, Flectobacillus | 0,0 | 0,4 | 0,0 | 0,0 | 0,0 | 0,0 | 0,0 |  |
| Bacteroidetes, Cytophagia, Cytophagales, Cytophagaceae, Hymenobacter | 3,2 | 0,0 | 48,6 | 34,8 | 35,2 | 26,6 | 21,7 |  |
| Bacteroidetes, Flavobacteriia, Flavobacteriales, Flavobacteriaceae, Flavobacterium | 0,0 | 5,2 | 0,0 | 0,0 | 0,0 | 0,0 | 0,0 |  |
| Bacteroidetes, Sphingobacteriia, Sphingobacteriales | 0,0 | 4,5 | 0,0 | 0,0 | 0,0 | 0,0 | 0,0 |  |
| Bacteroidetes, [Saprospirae], [Saprospirales], Chitinophagaceae | 0,0 | 0,0 | 0,0 | 0,0 | 0,0 | 0,6 | 1,2 |  |
| Bacteroidetes, [Saprospirae], [Saprospirales], Chitinophagaceae | 33,2 | 0,0 | 7,1 | 32,2 | 38,3 | 5,5 | 6,9 |  |
| Bacteroidetes, [Saprospirae], [Saprospirales], Saprospiraceae | 0,0 | 0,0 | 0,0 | 0,0 | 0,0 | 0,4 | 0,6 |  |
| Chloroflexi, Ktedonobacteria, Thermogemmatisporales, Thermogemmatisporaceae | 0,0 | 0,0 | 0,0 | 0,0 | 0,0 | 0,0 | 0,0 |  |
| Cyanobacteria | 0,0 | 0,0 | 0,0 | 0,0 | 0,0 | 0,0 | 0,0 |  |
| Cyanobacteria, Synechococcophycideae, Pseudanabaenales, Pseudanabaenaceae, Leptolyngbya | 0,0 | 0,0 | 0,0 | 0,0 | 0,0 | 9,5 | 9,4 |  |
| Cyanobacteria, Synechococcophycideae, Pseudanabaenales, Pseudanabaenaceae, Pseudanabaena | 0,0 | 0,0 | 0,0 | 0,0 | 0,0 | 0,0 | 0,0 |  |
| Cyanobacteria, Synechococcophycideae, Synechococcales, Chamaesiphonaceae | 0,0 | 0,0 | 0,0 | 0,0 | 0,0 | 6,7 | 11,0 |  |
| Firmicutes, Clostridia, Clostridiales, Clostridiaceae, Clostridium | 0,0 | 0,1 | 0,0 | 0,0 | 0,0 | 0,0 | 0,1 |  |
| GN02, BD1-5 | 0,0 | 4,7 | 0,0 | 0,0 | 0,0 | 0,0 | 0,0 |  |
| Proteobacteria, Alphaproteobacteria, Rhizobiales | 0,0 | 0,0 | 0,0 | 0,0 | 0,0 | 0,6 | 0,5 |  |
| Proteobacteria, Alphaproteobacteria, Rhizobiales, Methylocystaceae | 3,0 | 0,0 | 0,0 | 0,0 | 0,0 | 0,0 | 0,0 |  |
| Proteobacteria, Alphaproteobacteria, Rhizobiales, Rhizobiaceae | 0,0 | 0,0 | 0,0 | 0,0 | 0,0 | 0,0 | 0,0 |  |
| Proteobacteria, Alphaproteobacteria, Rhodospirillales, Acetobacteraceae | 3,8 | 0,5 | 0,0 | 0,0 | 0,7 | 0,0 | 0,2 |  |
| Proteobacteria, Alphaproteobacteria, Rhodospirillales, Acetobacteraceae, | 8,5 | 0,0 | 0,4 | 0,2 | 0,0 | 11,1 | 10,9 |  |
| Proteobacteria, Alphaproteobacteria, Sphingomonadales, Sphingomonadaceae | 0,0 | 0,0 | 0,0 | 0,0 | 0,0 | 0,8 | 0,8 |  |
| Proteobacteria, Alphaproteobacteria, Sphingomonadales, Sphingomonadaceae, | 0,0 | 0,0 | 0,0 | 0,0 | 0,0 | 0,0 | 0,0 |  |
| Proteobacteria, Alphaproteobacteria, Sphingomonadales, Sphingomonadaceae, Kaistobacter | 0,0 | 0,0 | 0,0 | 0,0 | 0,0 | 0,4 | 0,9 |  |
| Proteobacteria, Alphaproteobacteria, Sphingomonadales, Sphingomonadaceae, Novosphingobium | 0,0 | 0,0 | 0,0 | 0,0 | 0,0 | 0,0 | 0,0 |  |
| Proteobacteria, Alphaproteobacteria, Sphingomonadales, Sphingomonadaceae, Sphingomonas | 0,7 | 0,1 | 0,0 | 0,0 | 0,2 | 0,0 | 0,0 |  |
| Proteobacteria, Alphaproteobacteria, Sphingomonadales, Sphingomonadaceae, Zymomonas | 0,0 | 0,2 | 0,0 | 0,0 | 0,0 | 0,2 | 0,1 |  |
| Proteobacteria, Betaproteobacteria, Burkholderiales, Comamonadaceae | 0,8 | 0,0 | 0,7 | 1,0 | 0,0 | 0,4 | 0,4 |  |
| Proteobacteria, Betaproteobacteria, Burkholderiales, Comamonadaceae, Methylibium | 1,0 | 0,1 | 1,8 | 4,0 | 0,0 | 1,9 | 3,0 |  |
| Proteobacteria, Betaproteobacteria, Burkholderiales, Comamonadaceae, Polaromonas | 0,0 | 4,0 | 0,0 | 0,5 | 14,8 | 4,1 | 3,3 |  |
| Proteobacteria, Betaproteobacteria, Burkholderiales, Comamonadaceae, Rhodoferax | 0,0 | 0,1 | 0,0 | 0,0 | 0,0 | 0,0 | 0,0 |  |
| Proteobacteria, Betaproteobacteria, Burkholderiales, Comamonadaceae, Variovorax | 0,0 | 0,3 | 0,0 | 0,0 | 0,0 | 0,9 | 0,9 |  |
| Proteobacteria, Betaproteobacteria, Burkholderiales, Oxalobacteraceae | 8,2 | 0,0 | 1,3 | 0,6 | 0,6 | 0,0 | 0,0 |  |
| Proteobacteria, Betaproteobacteria, Burkholderiales, Oxalobacteraceae, Collimonas | 0,0 | 0,0 | 1,1 | 0,4 | 0,0 | 0,0 | 0,0 |  |
| Proteobacteria, Betaproteobacteria, Burkholderiales, Oxalobacteraceae, Herminiimonas | 16,7 | 58,0 | 3,3 | 6,0 | 9,9 | 0,8 | 0,5 |  |
| Proteobacteria, Betaproteobacteria, Burkholderiales, Oxalobacteraceae, Janthinobacterium | 0,0 | 21,5 | 0,0 | 0,0 | 0,3 | 0,0 | 0,0 |  |
| Proteobacteria, Betaproteobacteria, Ellin6067 | 0,0 | 0,0 | 0,0 | 0,0 | 0,0 | 1,9 | 2,2 |  |
| Proteobacteria, Gammaproteobacteria, Pseudomonadales, Pseudomonadaceae, Pseudomonas | 3,4 | 0,0 | 0,0 | 0,0 | 0,0 | 0,0 | 0,0 |  |
| Proteobacteria, Gammaproteobacteria, Xanthomonadales, Xanthomonadaceae, Rhodanobacter | 1,3 | 0,1 | 0,0 | 0,0 | 0,0 | 0,2 | 0,3 |  |
| TM7, TM7-3, EW055 | 0,0 | 0,0 | 0,0 | 0,0 | 0,0 | 0,0 | 0,4 |  |
| Verrucomicrobia, Verrucomicrobiae, Verrucomicrobiales, Verrucomicrobiaceae, | 0,0 | 0,0 | 0,0 | 0,0 | 0,0 | 0,0 | 0,0 |  |
| Verrucomicrobia, Verrucomicrobiae, Verrucomicrobiales, Verrucomicrobiaceae, Luteolibacter | 0,0 | 0,0 | 0,0 | 0,0 | 0,0 | 0,0 | 0,0 |  |
| WPS-2 | 0,0 | 0,0 | 0,0 | 0,0 | 0,0 | 3,2 | 3,4 |  |
| [Thermi], Deinococci, Deinococcales, Deinococcaceae, Deinococcus | 0,0 | 0,0 | 0,0 | 0,0 | 0,0 | 3,4 | 2,3 |  |
| Other | 0,1 | 0,0 | 0,0 | 0,0 | 0,0 | 0,0 | 0,0 |  |
|  |  |  |  |  |  |  |  |  |
|  | **WP212** | **GrIS16-10** | **GrIS16-5** | **MIT12-9** | **SVA13-19** | **SVA13-50** | **SVA13-8** | **TAR13-13** |
| Acidobacteria, Acidobacteriia, Acidobacteriales, Acidobacteriaceae | 0,5 | 15,6 | 3,6 | 3,6 | 15,8 | 0,0 | 2,2 | 10,3 |
| Acidobacteria, Acidobacteriia, Acidobacteriales, Acidobacteriaceae | 0,2 | 0,0 | 0,0 | 3,7 | 0,0 | 0,0 | 0,0 | 0,1 |
| Acidobacteria, Solibacteres, Solibacterales, Solibacteraceae, Candidatus Solibacter | 0,2 | 0,8 | 0,2 | 0,0 | 0,0 | 0,0 | 0,4 | 0,2 |
| Actinobacteria, Acidimicrobiia, Acidimicrobiales | 0,2 | 0,5 | 0,1 | 0,1 | 0,0 | 0,0 | 0,9 | 0,1 |
| Actinobacteria, Actinobacteria, Actinomycetales, ACK-M1 | 0,0 | 2,3 | 0,3 | 0,0 | 0,1 | 0,0 | 3,7 | 1,1 |
| Actinobacteria, Actinobacteria, Actinomycetales, Intrasporangiaceae | 1,9 | 0,0 | 0,0 | 0,3 | 0,0 | 0,0 | 0,4 | 0,0 |
| Actinobacteria, Actinobacteria, Actinomycetales, Microbacteriaceae, Salinibacterium | 5,2 | 5,5 | 0,4 | 4,6 | 0,2 | 0,6 | 9,5 | 4,1 |
| Actinobacteria, Actinobacteria, Actinomycetales, Nocardiaceae | 1,0 | 0,0 | 0,0 | 1,5 | 51,3 | 25,4 | 0,7 | 0,0 |
| Actinobacteria, Actinobacteria, Actinomycetales, Sporichthyaceae | 1,8 | 0,7 | 0,1 | 0,4 | 0,0 | 0,0 | 1,4 | 0,1 |
| Armatimonadetes, Armatimonadia, FW68 | 5,1 | 18,1 | 10,1 | 8,1 | 21,9 | 0,0 | 3,6 | 9,8 |
| Bacteroidetes | 11,6 | 16,1 | 6,4 | 6,8 | 1,5 | 0,0 | 4,2 | 17,4 |
| Bacteroidetes, Cytophagia, Cytophagales, Cytophagaceae, Flectobacillus | 0,1 | 0,0 | 0,0 | 0,0 | 0,0 | 0,0 | 0,7 | 0,0 |
| Bacteroidetes, Cytophagia, Cytophagales, Cytophagaceae, Hymenobacter | 11,8 | 6,3 | 5,5 | 14,4 | 0,1 | 0,0 | 3,8 | 18,6 |
| Bacteroidetes, Flavobacteriia, Flavobacteriales, Flavobacteriaceae, Flavobacterium | 0,0 | 0,0 | 0,0 | 0,0 | 0,0 | 0,0 | 0,0 | 0,0 |
| Bacteroidetes, Sphingobacteriia, Sphingobacteriales | 0,0 | 0,0 | 0,0 | 0,0 | 0,0 | 0,0 | 0,0 | 0,0 |
| Bacteroidetes, [Saprospirae], [Saprospirales], Chitinophagaceae | 2,0 | 1,9 | 0,1 | 0,1 | 0,0 | 0,0 | 2,4 | 0,2 |
| Bacteroidetes, [Saprospirae], [Saprospirales], Chitinophagaceae | 14,3 | 0,0 | 0,1 | 1,1 | 0,0 | 0,0 | 1,6 | 0,5 |
| Bacteroidetes, [Saprospirae], [Saprospirales], Saprospiraceae | 0,7 | 0,0 | 0,5 | 0,0 | 0,0 | 0,0 | 4,9 | 0,2 |
| Chloroflexi, Ktedonobacteria, Thermogemmatisporales, Thermogemmatisporaceae | 0,0 | 0,0 | 22,8 | 0,0 | 0,0 | 0,0 | 1,3 | 0,0 |
| Cyanobacteria | 0,0 | 0,1 | 13,4 | 0,0 | 0,0 | 0,0 | 0,0 | 0,0 |
| Cyanobacteria, Synechococcophycideae, Pseudanabaenales, Pseudanabaenaceae, Leptolyngbya | 3,7 | 0,4 | 6,8 | 0,3 | 0,7 | 0,0 | 27,5 | 0,4 |
| Cyanobacteria, Synechococcophycideae, Pseudanabaenales, Pseudanabaenaceae, Pseudanabaena | 0,0 | 0,0 | 0,0 | 0,0 | 0,0 | 0,0 | 6,8 | 0,0 |
| Cyanobacteria, Synechococcophycideae, Synechococcales, Chamaesiphonaceae | 1,5 | 2,2 | 2,2 | 0,0 | 0,0 | 0,0 | 0,6 | 0,2 |
| Firmicutes, Clostridia, Clostridiales, Clostridiaceae, Clostridium | 0,1 | 0,1 | 0,0 | 1,2 | 0,0 | 0,0 | 0,3 | 0,1 |
| GN02, BD1-5 | 0,0 | 0,0 | 0,0 | 0,0 | 0,0 | 0,0 | 0,0 | 0,0 |
| Proteobacteria, Alphaproteobacteria, Rhizobiales | 1,2 | 0,0 | 0,0 | 0,0 | 0,0 | 0,0 | 0,2 | 0,1 |
| Proteobacteria, Alphaproteobacteria, Rhizobiales, Methylocystaceae | 0,0 | 0,0 | 0,0 | 0,0 | 0,0 | 0,0 | 0,0 | 0,0 |
| Proteobacteria, Alphaproteobacteria, Rhizobiales, Rhizobiaceae | 0,0 | 0,0 | 0,0 | 0,0 | 0,0 | 0,0 | 0,0 | 0,0 |
| Proteobacteria, Alphaproteobacteria, Rhodospirillales, Acetobacteraceae | 0,2 | 0,0 | 0,0 | 2,0 | 0,1 | 0,0 | 1,2 | 0,3 |
| Proteobacteria, Alphaproteobacteria, Rhodospirillales, Acetobacteraceae, | 13,3 | 13,7 | 20,8 | 11,8 | 2,8 | 67,6 | 8,2 | 21,3 |
| Proteobacteria, Alphaproteobacteria, Sphingomonadales, Sphingomonadaceae | 1,9 | 2,9 | 0,9 | 2,1 | 0,3 | 0,0 | 1,3 | 5,9 |
| Proteobacteria, Alphaproteobacteria, Sphingomonadales, Sphingomonadaceae, | 0,0 | 1,2 | 2,2 | 0,0 | 0,0 | 0,0 | 0,3 | 0,1 |
| Proteobacteria, Alphaproteobacteria, Sphingomonadales, Sphingomonadaceae, Kaistobacter | 0,8 | 0,0 | 0,0 | 0,0 | 0,0 | 0,0 | 0,4 | 0,0 |
| Proteobacteria, Alphaproteobacteria, Sphingomonadales, Sphingomonadaceae, Novosphingobium | 0,0 | 0,0 | 0,0 | 0,0 | 0,0 | 4,3 | 0,0 | 0,0 |
| Proteobacteria, Alphaproteobacteria, Sphingomonadales, Sphingomonadaceae, Sphingomonas | 0,3 | 0,0 | 0,0 | 0,0 | 0,0 | 0,0 | 0,1 | 0,0 |
| Proteobacteria, Alphaproteobacteria, Sphingomonadales, Sphingomonadaceae, Zymomonas | 0,5 | 0,1 | 0,6 | 0,0 | 0,0 | 0,0 | 0,7 | 0,1 |
| Proteobacteria, Betaproteobacteria, Burkholderiales, Comamonadaceae | 0,3 | 0,1 | 0,1 | 1,8 | 0,0 | 0,0 | 0,1 | 0,6 |
| Proteobacteria, Betaproteobacteria, Burkholderiales, Comamonadaceae, Methylibium | 3,0 | 0,0 | 0,0 | 0,3 | 0,0 | 0,0 | 0,0 | 0,0 |
| Proteobacteria, Betaproteobacteria, Burkholderiales, Comamonadaceae, Polaromonas | 7,5 | 0,3 | 0,5 | 0,5 | 0,0 | 0,0 | 1,2 | 0,3 |
| Proteobacteria, Betaproteobacteria, Burkholderiales, Comamonadaceae, Rhodoferax | 0,0 | 0,0 | 0,0 | 0,0 | 0,0 | 0,0 | 0,0 | 0,0 |
| Proteobacteria, Betaproteobacteria, Burkholderiales, Comamonadaceae, Variovorax | 1,4 | 0,2 | 0,0 | 3,4 | 0,0 | 0,0 | 0,0 | 1,1 |
| Proteobacteria, Betaproteobacteria, Burkholderiales, Oxalobacteraceae | 0,0 | 0,0 | 0,0 | 0,5 | 0,0 | 0,0 | 0,0 | 0,0 |
| Proteobacteria, Betaproteobacteria, Burkholderiales, Oxalobacteraceae, Collimonas | 0,0 | 0,0 | 0,0 | 0,0 | 0,0 | 0,0 | 0,0 | 0,0 |
| Proteobacteria, Betaproteobacteria, Burkholderiales, Oxalobacteraceae, Herminiimonas | 0,6 | 0,0 | 0,0 | 0,5 | 0,0 | 0,0 | 0,6 | 0,1 |
| Proteobacteria, Betaproteobacteria, Burkholderiales, Oxalobacteraceae, Janthinobacterium | 0,0 | 0,0 | 0,0 | 0,0 | 0,0 | 0,0 | 0,0 | 0,0 |
| Proteobacteria, Betaproteobacteria, Ellin6067 | 0,7 | 0,0 | 0,0 | 0,2 | 0,0 | 0,0 | 0,3 | 0,0 |
| Proteobacteria, Gammaproteobacteria, Pseudomonadales, Pseudomonadaceae, Pseudomonas | 0,0 | 0,0 | 0,0 | 0,0 | 0,0 | 0,0 | 0,0 | 0,0 |
| Proteobacteria, Gammaproteobacteria, Xanthomonadales, Xanthomonadaceae, Rhodanobacter | 0,2 | 0,0 | 0,0 | 6,5 | 0,0 | 0,0 | 0,1 | 2,3 |
| TM7, TM7-3, EW055 | 1,2 | 0,7 | 0,0 | 0,5 | 3,3 | 0,0 | 0,7 | 0,5 |
| Verrucomicrobia, Verrucomicrobiae, Verrucomicrobiales, Verrucomicrobiaceae, | 0,0 | 0,0 | 0,0 | 0,0 | 0,0 | 0,0 | 0,0 | 0,0 |
| Verrucomicrobia, Verrucomicrobiae, Verrucomicrobiales, Verrucomicrobiaceae, Luteolibacter | 0,0 | 0,0 | 0,0 | 0,0 | 0,0 | 0,0 | 0,0 | 0,0 |
| WPS-2 | 3,8 | 10,0 | 2,2 | 23,5 | 0,2 | 0,0 | 4,8 | 3,9 |
| [Thermi], Deinococci, Deinococcales, Deinococcaceae, Deinococcus | 1,0 | 0,0 | 0,0 | 0,4 | 0,6 | 0,0 | 2,4 | 0,0 |
| Other | 0,0 | 0,0 | 0,0 | 0,0 | 1,0 | 1,9 | 0,0 | 0,0 |
|  |  |  |  |  |  |  |  |  |

**Supplementary Table 3.** Cryoflora community structure, based on the **18S rRNA gene**  data set, comprising the 51 most abundant ASVs. An identity threshold of ~99.4% had to be passed in order to be considered as a database match (i.e., maximally 2 bp nucleotide difference in a 342 bp sequence was allowed; Lutz et al. 2019). Sequences below this threshold were recorded as “no blast hit”.

|  |  | **SNOW** | | | | | | **ICE** | | | | | | | | | | | |
| --- | --- | --- | --- | --- | --- | --- | --- | --- | --- | --- | --- | --- | --- | --- | --- | --- | --- | --- | --- |
| **ASV ID** | **Assignment** | **WP117** | **WP119** | **WP127** | **WP181** | **WP199** | **WP203** | **WP205** | **WP165** | **WP166** | **WP212** | **GrIS16-10** | **GrIS16-5** | **GrIS16-9** | **MIT12-9** | **SVA13-19** | **SVA13-50** | **SVA13-8** | **TAR13-13** |
| 108f173c313c470ccdad3d9f1c58c10f | *Ancylonema nordenskioeldii* WP211 MW922838.1 | 0.0 | 0.0 | 0.0 | 0.1 | 0.0 | 0.0 | 0.0 | 0.1 | 0.2 | 32.1 | 64.6 | 32.1 | 89.3 | 3.2 | 14.9 | 22.1 | 5.2 | 7.4 |
| 4f5e543583c95f557e91a59adb4677c4 | *Ancylonema nordenskioeldii* WP211 MW922838.1 | 0.0 | 0.0 | 0.2 | 0.1 | 0.0 | 0.0 | 0.0 | 1.9 | 2.0 | 17.8 | 12.3 | 60.4 | 9.3 | 0.6 | 19.8 | 22.1 | 2.4 | 57.3 |
| 7e5e25b3d419e231973472b0bf4ce832 | no blast hit**^1^** | 0.0 | 0.0 | 0.0 | 0.0 | 0.0 | 0.0 | 0.0 | 0.6 | 0.5 | 0.2 | 0.0 | 0.0 | 0.0 | 0.1 | 0.0 | 0.0 | 0.0 | 0.0 |
| 3c252251e99e7e8c84114b5e1b8be176 | *Ancylonema nordenskioeldii* WP211 MW922838.1 | 0.0 | 0.0 | 0.0 | 0.0 | 0.0 | 0.0 | 0.0 | 0.0 | 0.0 | 0.0 | 0.0 | 0.0 | 0.0 | 0.0 | 0.0 | 1.1 | 0.0 | 0.0 |
| cdd18738e2f0a7a837e56b17d4d6e958 | *Ancylonema nordenskioeldii* WP211 MW922838.1 | 0.0 | 0.0 | 0.0 | 0.0 | 0.0 | 0.0 | 0.0 | 0.0 | 0.0 | 0.0 | 0.0 | 0.0 | 0.0 | 0.0 | 1.1 | 0.0 | 0.0 | 0.0 |
| 01d988174f2e8cf329e9b70cb45b23c8 | *Ancylonema alaskanum* CCCryo BS_0002-2009 JF430424.1 | 0.0 | 0.0 | 0.0 | 0.0 | 0.0 | 0.0 | 0.0 | 80.4 | 74.7 | 34.2 | 0.0 | 0.0 | 0.0 | 15.1 | 16.3 | 0.9 | 7.3 | 4.2 |
| b938ff6c74dbf241f39b1ec6808e9dab | no blast hit**^2^** | 0.0 | 0.0 | 0.0 | 0.0 | 0.0 | 0.0 | 0.0 | 0.0 | 0.0 | 0.0 | 0.0 | 0.3 | 0.0 | 0.0 | 0.0 | 0.0 | 6.9 | 0.0 |
| 3c5a37c63a01cd98e1a4991b819d32f5 | uncultured alga OTU008 LC371432.1, *Chlainomonas* sp. Bagley_NODE_5578 OP003985.1 | 0.0 | 0.0 | 0.0 | 0.0 | 0.0 | 0.0 | 0.2 | 1.0 | 1.1 | 0.2 | 0.2 | 2.4 | 0.2 | 0.3 | 0.6 | 0.0 | 4.0 | 5.1 |
| 6091db82655cca1c1f4272894fb58721 | *Chloromonas nivalis* P24/DR4 (GU117576.1). *Chloromonas nivalis* subsp. *tatrae* LP01 (KY499614.1) | 0.0 | 0.0 | 0.0 | 0.0 | 0.6 | 0.0 | 0.2 | 0.2 | 0.2 | 0.1 | 0.0 | 0.0 | 0.0 | 2.8 | 0.0 | 0.0 | 0.0 | 0.0 |
| 1d4c45ab3818daad0c5aa0ac61e793a7 | *Chlamydomonas* sp. PA-1 (AB902973.1). uncultured alga OTU001 (LC371408) | 0.0 | 0.0 | 0.0 | 8.1 | 0.9 | 0.0 | 0.0 | 0.0 | 0.1 | 0.0 | 0.0 | 0.0 | 0.0 | 2.4 | 0.0 | 0.0 | 0.0 | 0.0 |
| 3f085ef1439fc6ea3e704c6de70b8bee | *Sanguina nivaloides* CCCryo RS_0015-2010 (JQ790560.1), *Sanguina aurantia* CCCryo RS_0017-2010 (MK728645.1) | 81.1 | 0.3 | 24.1 | 80.7 | 97.0 | 0.3 | 84.9 | 0.0 | 0.0 | 1.1 | 0.6 | 0.1 | 0.0 | 20.1 | 21.5 | 17.4 | 1.4 | 0.6 |
| b76ddc20ef6fcd1d8b6fff396d5b5bb0 | *Sanguina nivaloides* CCCryo RS_0011 (MK728642.1) | 0.0 | 0.0 | 68.1 | 0.0 | 0.0 | 0.0 | 7.9 | 0.0 | 0.0 | 0.0 | 0.0 | 0.0 | 0.0 | 0.0 | 0.0 | 0.8 | 0.0 | 0.2 |
| ac8563018cf97b5404b22c87fc7e6178 | *Sanguina nivaloides* CCCryo RS_0015-2010 (JQ790560.1), *Sanguina aurantia* CCCryo RS_0017-2010 (MK728645.1) | 0.0 | 0.0 | 0.0 | 0.0 | 0.0 | 0.0 | 0.0 | 0.0 | 0.0 | 0.0 | 0.0 | 0.0 | 0.0 | 1.1 | 12.0 | 1.8 | 1.5 | 0.0 |
| 1f969e2be5cefdd668921bdcb91a1fc2 | Uncultured eukaryote clone Kili_10G_N7_18S_p1 (KX771795.1) | 5.2 | 0.0 | 0.9 | 0.5 | 0.0 | 0.0 | 0.0 | 0.0 | 0.0 | 0.3 | 0.0 | 0.0 | 0.0 | 0.0 | 0.0 | 0.0 | 0.0 | 0.0 |
| 4c517a8ad8fa7a4a429a305eefc1480a | Uncultured eukaryote clone elb6-t5-otu1 (GU117581.1) | 0.0 | 0.0 | 0.0 | 0.0 | 0.0 | 0.0 | 0.0 | 0.0 | 0.0 | 0.0 | 0.0 | 0.0 | 0.0 | 6.2 | 0.2 | 0.0 | 0.0 | 0.0 |
| 768cb0fdc32c240b44d6567bb5548bf9 | no blast hit**^3^** | 0.0 | 0.0 | 0.0 | 0.0 | 0.0 | 0.0 | 0.0 | 0.0 | 0.0 | 0.0 | 0.0 | 0.0 | 0.0 | 0.0 | 0.0 | 2.4 | 0.0 | 0.0 |
| fdddb545da49d0392bf9f3aef80c683a | *Limnomonas svalbardensis* CCCryo 217-05 (GU117581.1) | 0.0 | 0.0 | 0.0 | 0.0 | 0.0 | 0.0 | 0.0 | 0.0 | 0.0 | 0.0 | 0.0 | 0.0 | 0.0 | 0.0 | 0.0 | 8.6 | 0.0 | 0.0 |
| 4d1aac2588a6d5b780f5785cd3c16558 | *Limnomonas svalbardensis* CCCryo 217-05 (GU117581.1) | 0.0 | 0.0 | 0.0 | 0.0 | 0.0 | 0.0 | 0.0 | 0.0 | 0.0 | 0.0 | 0.0 | 0.0 | 0.0 | 0.0 | 0.0 | 1.0 | 0.0 | 0.0 |
| 3a6f74846a259c3fe4b782696e501d10 | *Chloroidium antarcticum* ISBAL-1013 (MH551519.1) | 0.0 | 0.0 | 0.0 | 0.0 | 0.0 | 0.0 | 0.0 | 0.0 | 0.0 | 0.0 | 0.0 | 0.0 | 0.0 | 0.1 | 0.0 | 0.0 | 0.0 | 0.0 |
| 69c6dec1deb2857887216c7755d301f7 | *Chloromonas muramotoi* HkCl-57 (LC438435.1) | 0.0 | 0.6 | 0.0 | 0.0 | 0.0 | 0.0 | 0.0 | 0.0 | 0.0 | 0.1 | 0.0 | 0.0 | 0.0 | 3.8 | 0.0 | 0.0 | 0.0 | 0.3 |
| 9a733580b2bc95b6819f41dbc6cbf5ae | *Chlorominima collina* CCAP 6/01 (MW553075) | 0.0 | 36.4 | 0.0 | 0.0 | 0.0 | 0.0 | 0.0 | 0.0 | 0.0 | 0.0 | 0.0 | 0.0 | 0.0 | 0.0 | 0.0 | 0.0 | 0.0 | 0.0 |
| c52a8d1b7f8c371ae83e862b5e4ce4df | *Chloromonas platystigma* CCCryo 020-99 (AF514401.1) | 0.0 | 0.0 | 0.0 | 0.0 | 0.0 | 0.0 | 0.0 | 0.2 | 0.2 | 0.6 | 0.0 | 0.0 | 0.0 | 0.0 | 0.0 | 0.0 | 0.0 | 0.0 |
| 8e78dd8be1307ab330f601796a8d5eba | *Chloromonas miwae* NIES-2379 (AB906350.1) | 0.1 | 0.0 | 0.0 | 0.0 | 0.0 | 0.0 | 0.0 | 0.0 | 0.0 | 0.0 | 0.0 | 0.0 | 0.0 | 0.0 | 0.0 | 0.0 | 0.0 | 0.0 |
| 8b85c6fd6d8aaa125d6a20af085760f1 | no blast hit**^4^** | 0.1 | 0.0 | 0.5 | 0.0 | 0.0 | 0.0 | 0.0 | 0.0 | 0.0 | 0.0 | 0.0 | 0.0 | 0.0 | 0.0 | 0.0 | 0.0 | 0.0 | 0.0 |
| 9644d041c52e46d39f6373bf5d1fd78a | Uncultured alga OTU025 (LC371427). uncultured alga (LC371425) | 0.0 | 0.0 | 0.0 | 0.0 | 0.0 | 0.0 | 0.1 | 0.0 | 0.0 | 0.0 | 0.0 | 0.0 | 0.0 | 0.0 | 0.0 | 0.0 | 0.0 | 0.0 |
| cce46e8225b5f705977aa20f2aa12a9e | *Chloromonas brevispina* K-2 (MG791867), *Chloromonas brevispina* Hakkoda-1 (LC012710), *Chloromonas krienitzii* Gassan-A (LC012709) | 0.0 | 0.0 | 0.0 | 0.1 | 0.0 | 0.0 | 0.0 | 0.0 | 0.0 | 0.2 | 0.0 | 0.0 | 0.0 | 0.3 | 0.0 | 0.0 | 0.0 | 0.2 |
| 28d008b8aca181f3d52193111883941f | *Chloromonas hindakii* WP129/CCCryo 531-19 (MN251865.1), *Chloromonas nivalis* Gassan-B (LC012714.1), *Chloromonas polyptera* (JQ790556.1) etc. | 0.0 | 0.0 | 1.8 | 0.3 | 0.1 | 0.0 | 0.3 | 0.0 | 0.0 | 0.1 | 0.0 | 0.0 | 0.0 | 2.6 | 0.0 | 2.3 | 12.7 | 0.1 |
| ba4ec92049883a3a0992b19743a3b79e | Uncultured alga OTU009 (LC371415.1), uncultured alga isolate 0935-5 (LC371440.1), etc. | 2.2 | 0.0 | 3.0 | 7.0 | 0.0 | 0.0 | 0.1 | 0.0 | 0.0 | 0.0 | 0.0 | 0.0 | 0.0 | 0.0 | 0.0 | 0.0 | 0.0 | 0.0 |
| 598c14014d4765124454c64bfba4f62b | Uncultured alga OTU003 (LC371418.1) | 0.0 | 0.0 | 0.2 | 0.0 | 0.0 | 0.0 | 0.5 | 0.0 | 0.0 | 0.4 | 0.0 | 0.0 | 0.0 | 1.2 | 0.0 | 0.0 | 0.0 | 1.0 |
| d0dba359aef0007cd58ffddb5f6cb730 | uncultured alga OTU009 (LC371415.1). uncultured alga isolate 0935-5 (LC371440.1), uncultured *Chloromonas* sp. AL4 (AB903027.1) etc. | 0.0 | 0.0 | 0.0 | 0.0 | 0.0 | 0.0 | 3.4 | 0.0 | 0.0 | 0.0 | 0.0 | 0.0 | 0.0 | 0.0 | 0.0 | 0.0 | 0.0 | 0.0 |
| 407c4e26ebecda896fb7c81da677baef | Uncultured alga OTU003 (LC371418.1) | 1.6 | 0.0 | 0.0 | 0.0 | 0.0 | 0.0 | 0.0 | 0.0 | 0.0 | 0.0 | 0.0 | 0.0 | 0.0 | 0.0 | 0.0 | 0.0 | 0.0 | 0.0 |
| 488a42ea193343f32dfbc5b3ebe6807d | *Chloromonas muramotoi* HkCl-57 (LC438435.1), *Chloromonas platystigma* CCCryo 020-99 (AF514401.1) | 0.0 | 0.0 | 0.0 | 2.0 | 0.0 | 0.0 | 0.0 | 0.0 | 0.0 | 0.1 | 0.0 | 0.0 | 0.0 | 0.0 | 0.0 | 0.0 | 0.0 | 0.0 |
| 2b2f2404faef7274fe24714a64cd14b3 | no blast hit**^5^** | 0.0 | 0.0 | 0.0 | 0.0 | 0.0 | 0.0 | 0.0 | 0.0 | 0.0 | 0.0 | 0.0 | 0.0 | 0.0 | 0.0 | 1.2 | 0.0 | 0.0 | 0.0 |
| d69e17b6f9ab4590d02280b69065435a | *Koliellopsis inundata* strain FACHB-2451 (MT274431.1) | 0.0 | 58.1 | 0.0 | 0.0 | 0.0 | 0.0 | 0.0 | 0.4 | 0.4 | 0.0 | 0.0 | 0.0 | 0.0 | 0.6 | 0.0 | 1.1 | 6.9 | 4.0 |
| aa664f775cad8f4e42f68519713d0241 | *Raphidonema sempervirens* KMY-2018 SR1-B (MK2627871) | 0.0 | 0.0 | 0.1 | 0.0 | 0.1 | 0.0 | 0.1 | 5.0 | 6.4 | 0.7 | 2.4 | 0.4 | 0.0 | 24.4 | 1.1 | 1.5 | 9.6 | 5.7 |
| 4891711e29d84b4a6a25caed2f760c18 | Uncultured eukaryote clone TE107A (KM870650.1) | 0.0 | 0.0 | 0.0 | 0.0 | 0.0 | 0.0 | 0.0 | 0.3 | 0.8 | 0.0 | 13.3 | 2.3 | 0.0 | 6.3 | 0.2 | 0.0 | 1.4 | 7.3 |
| 091298bf0b7c04d20f27ab641c13cb94 | *Koliellopsis inundata* strain FACHB-2451 (MT274431.1)*, Raphidonema sempervirens* KMY-2018 SR1-B (MK262787.1), *Raphidonema sempervirens* KMY-2018 SR1-A (MK262786.1), *Raphidonema sempervirens* KMY-2018 GR1-G (MK262785.1), etc. | 0.1 | 0.0 | 0.0 | 0.0 | 0.0 | 0.0 | 0.0 | 3.1 | 3.9 | 8.8 | 0.0 | 0.0 | 0.1 | 0.6 | 0.0 | 0.0 | 0.2 | 2.1 |
| b49b837b27d987c16539e8dcceb92f2a | Uncultured eukaryote clone TE203C (KM870706.1) | 0.0 | 0.0 | 0.0 | 0.0 | 0.0 | 0.0 | 0.0 | 0.5 | 1.1 | 0.3 | 4.2 | 0.7 | 0.0 | 0.0 | 0.0 | 0.0 | 0.0 | 0.0 |
| ab587b0fbe9a3cab6366e8e85d6d54d5 | Uncultured eukaryote clone TE107B (KM870651.1), Uncultured eukaryote clone TE101H (KM870611.1) | 0.0 | 0.0 | 0.0 | 0.0 | 0.0 | 0.0 | 0.0 | 0.9 | 1.7 | 0.3 | 0.0 | 0.0 | 0.0 | 2.5 | 0.0 | 0.0 | 0.0 | 2.2 |
| 5ed705bc5bc3a38a9e3f024d89adc8a1 | Uncultured eukaryote clone TE107B (KM870651.1), Uncultured eukaryote clone TE101H (KM870611.1) | 0.0 | 0.0 | 0.0 | 0.0 | 0.0 | 0.0 | 0.0 | 0.0 | 0.0 | 0.0 | 0.0 | 0.3 | 0.0 | 0.0 | 0.0 | 0.0 | 3.3 | 0.0 |
| 2ad4f574bf7cfd93ee1d8daf1a901e1f | no blast hit**^6^** | 0.0 | 0.0 | 0.0 | 0.0 | 0.0 | 0.0 | 0.0 | 0.0 | 0.0 | 0.0 | 0.0 | 0.0 | 0.0 | 0.0 | 0.0 | 0.0 | 0.0 | 0.0 |
| a87fba4ddc54323be444ccefe8ca866e | *Trebouxia jamesii* UBT-86.132E2 (Z68700.1) | 3.8 | 0.0 | 0.0 | 0.0 | 0.0 | 0.0 | 0.0 | 0.0 | 0.0 | 0.0 | 0.0 | 0.0 | 0.0 | 0.5 | 0.0 | 0.0 | 0.0 | 0.0 |
| 8d7e6bd6ecf6ff9661b2084024d18348 | no blast hit**^7^** | 0.1 | 0.0 | 0.7 | 0.0 | 0.2 | 0.0 | 0.0 | 3.9 | 4.4 | 1.1 | 0.0 | 0.0 | 0.0 | 0.0 | 0.0 | 0.0 | 0.0 | 0.0 |
| 6789bb8bc412b57e9f3645f055441589 | *Hydrurus* sp. Esp21 (MG674912.1), Chrysophyceae StChRivDWTP17 (MK618749.1). eukaryote 44c*_*37218 (KT815033.1) | 0.0 | 0.0 | 0.0 | 0.0 | 0.1 | 28.5 | 0.1 | 0.0 | 0.0 | 0.0 | 0.0 | 0.0 | 0.0 | 0.0 | 0.0 | 0.0 | 0.4 | 0.0 |
| 1c446bd46681b288ca51c493473c9e69 | *Hydrurus* sp. Sva 10-3 (HE820740.1) | 0.0 | 0.0 | 0.0 | 0.0 | 0.2 | 64.8 | 0.0 | 0.0 | 0.0 | 0.0 | 0.0 | 0.0 | 0.0 | 0.0 | 0.0 | 0.0 | 0.0 | 0.0 |
| 79d7ef14545b4ade9dd6f1395d834032 | Hydrurus sp. Sva 10-3 (HE820740.1) | 0.0 | 0.0 | 0.0 | 0.0 | 0.0 | 2.1 | 0.0 | 0.0 | 0.0 | 0.0 | 0.0 | 0.0 | 0.0 | 0.0 | 0.0 | 0.0 | 0.0 | 0.0 |
| c90a5d8064cc46a79886831f04747705 | *Hydrurus* sp. Sva 10-3 (HE820740.1) | 0.0 | 0.0 | 0.0 | 0.0 | 0.0 | 1.0 | 0.0 | 0.0 | 0.0 | 0.0 | 0.0 | 0.0 | 0.0 | 0.0 | 0.0 | 0.0 | 0.0 | 0.0 |
| 29bf5087ca2e9adee02d446e1e0dcff7 | *Hydrurus* sp. Sva 10-3 (HE820740.1) | 0.0 | 0.0 | 0.0 | 0.0 | 0.0 | 0.7 | 0.0 | 0.0 | 0.0 | 0.0 | 0.0 | 0.0 | 0.0 | 0.0 | 0.0 | 0.0 | 0.0 | 0.0 |
| 9a02cd29a90e36a21765aa2703e923b1 | *Ochromonas* clone Esp29 (MG674914), Uncultured stramenopile clone 7-B12 (FN690697), Uncultured eukaryote clone AMD_18S_OTUa_1072 (MK003353) etc. | 0.0 | 0.0 | 0.0 | 0.0 | 0.0 | 0.0 | 0.0 | 0.0 | 0.0 | 0.0 | 0.0 | 0.0 | 0.0 | 0.3 | 0.0 | 0.0 | 30.6 | 0.0 |
| 717e00e8877a8e315ea9193bcd9b5858 | *Botrydiopsis constricta* (AJ579339.1) | 0.0 | 0.0 | 0.0 | 0.0 | 0.0 | 0.0 | 0.0 | 0.0 | 0.0 | 0.0 | 0.0 | 0.0 | 0.0 | 0.0 | 0.0 | 0.0 | 0.7 | 0.0 |
| fb2134968c8079550185baf6966d34f3 | no blast hit**^8^** | 0.0 | 0.0 | 0.0 | 0.0 | 0.0 | 0.0 | 0.0 | 0.0 | 0.0 | 0.0 | 0.1 | 0.0 | 0.0 | 0.0 | 0.0 | 0.0 | 0.2 | 1.0 |
| Other |  | 5.7 | 4.6 | 0.5 | 1.0 | 0.7 | 2.6 | 2.1 | 1.5 | 2.1 | 1.3 | 2.3 | 1.1 | 1.0 | 4.7 | 11.1 | 16.8 | 5.0 | 1.3 |

^1^3 bp difference (99.1% identity) to *Ancylonema nordenskioeldii* WP211 (MW922838.1)

^2^15 bp difference (95.4% identity) to *Staurodesmus mucronatus* M 1394 (AJ428103.1)

^3^3 bp difference (99.1% identity) to Chlorophyta sp. I-155 clone A1 (EF432529.1), Uncultured eukaryote clone AMD_18S_OTUa_5283 (MK003890.1)

^4^4 bp difference (98.76% identity) to *Chloromonas nivalis* UTEX SNO71 (LC360465.1), *Chloromonas tenuis* UTEX SNO132 (AB906347.1), *Chloromonas hohamii* (UTEX SNO67) and others

^5^3 bp (99.1% identity) to Uncultured *Apatococcus* clone AEW7R_K37 (KP081319.1), Uncultured Trebouxiophyceae clone LSY2043 (MG493360.1) etc.

|  |
| --- |

^6^3 bp difference (99.1% identity) to *Stichococcus bacillaris* SAG 56.91 (MT078155.1), *Stichococcus bacillaris* SAG 335-8 (MT078154.1) and *Stichococcus bacillaris* WB74 (KF144232.1)

^7^10 bp difference (96.95% identity) to *Hydrurus foetidus* (FM955256.1), Uncultured Hydrurus isolate Sva 10_3 (HE820740.1) etc.

**^8^**10 bp difference (96.95% identity) to Uncultured stramenopile clone B1_4_1E_132 (JQ627411.1), Uncultured stramenopile clone RP2_6_1E_12 (JQ627410.1)

**Supplementary Table 4.** Cryoflora community structure, based on the **ITS2** data set (amplified by the “snow“ algal primers), comprising the 44 most abundant ASVs. An identity threshold of ~89% against a reference had to be passed in order to be considered as a database match. Sequences below this threshold were recorded as “no blast hit”. In the next step, compensatory base change (CBC) in homologous base pair positions in ITS2 were identified as a proxy of species identification. Unless ASVID was 100% identical with the reference, CBC search was conducted and it was done in the entire ITS2 secondary structure (see **Supplementary Figures 1-2** and **Supplementary Figures** **7-15** [column: SF]). Unless a CBC was found, the ASVID was assigned to the reference. If a CBC was found, the sequence was delimited as “no blast hit”.

| **ASV ID** | **Assignment** | **SF** | **WP**  **117** | **WP**  **119** | **WP**  **127** | **WP**  **181** | **WP**  **199** | **WP**  **203** | **WP**  **205** | **WP**  **212** | **GrIS**  **16-10** | **GrIS**  **16-5** | **GrIS**  **16-9** | **MIT**  **12-9** | **SVA**  **13-19** | **SVA**  **13-50** | **SVA**  **13-8** | **TAR**  **13-13** |
| --- | --- | --- | --- | --- | --- | --- | --- | --- | --- | --- | --- | --- | --- | --- | --- | --- | --- | --- |
| 81f5ec709b7448ed342d146c70fe8f49 | no blast hit^1^ | 10 | 0 | 0 | 11.2 | 0 | 0 | 0 | 0 | 0 | 0 | 0 | 0 | 0 | 0 | 0 | 0 | 0 |
| befe347a0eb994f55693cc01c6f0bc90 | uncultured Chlorophyta clone ALBC6 (JX435348) | 11 | 0 | 0 | 22.4 | 0 | 0 | 0 | 0 | 0 | 0 | 0 | 0 | 0 | 0 | 0 | 0 | 0 |
| 6e1818ae977266295a32e31102b53d80 | *Sanguina nivaloides* CCCryo 0015-2010 (MK728599) | 1-2 | 0 | 0 | 0 | 6.1 | 0 | 0 | 0 | 0 | 0 | 0 | 0 | 0 | 0 | 0 | 0 | 0 |
| 28f2b3f21bed71b8dc2196a80189ff33 | *Limnomonas svalbardensis* CCCryo 217-05 (GU117581) | 13 | 0 | 0 | 0 | 0 | 0 | 0.6 | 0 | 0 | 0 | 0 | 0 | 6.3 | 0 | 0 | 0 | 0 |
| 16a94e1df7d3bce5f14dbb669b335768 | no blast hit | -- | 0 | 0 | 22.9 | 0 | 0 | 0 | 0 | 0 | 0 | 0 | 0 | 0 | 0 | 0 | 0 | 0 |
| 20ce236c0ec95982dcb59eafed4bf026 | S*anguina* sp. DR74a_Trochiscia-type (OL962698) | 1-2 | 0 | 0 | 0 | 0 | 0 | 38 | 62.2 | 0 | 0 | 0 | 0 | 6.8 | 0 | 0 | 0 | 5.7 |
| 399608d04f0c5a06160d54d657ac40fe | *Sanguina aurantia* CCCryo RS_0017-2010 (MK728634) | 1-2 | 0 | 0 | 0 | 0 | 2.3 | 9.9 | 0 | 0 | 0 | 0 | 0 | 49.3 | 8.3 | 26.7 | 0 | 0 |
| 4985407c121d5195bcb5685fbb9e2e30 | *Sanguina* sp. DR74a_Trochiscia-type (OL962698) | 1-2 | 6.7 | 0 | 0 | 0 | 0 | 0 | 0 | 0 | 0 | 0 | 0 | 0 | 0 | 0 | 0 | 0 |
| 505d0a0b136693647e331821956c14d3 | *Sanguina aurantia* CCCryo RS_0020-2010 (MK728633) | 1-2 | 0 | 0 | 0 | 0 | 97.6 | 44.1 | 0 | 0 | 81 | 17 | 21.2 | 16.2 | 4 | 7.3 | 0 | 21.7 |
| 586563f91f085f301a20dd6e8051db61 | *Sanguina aurantia* CCCryo RS_0017-2010 (MK728634) | 1-2 | 0 | 0 | 0 | 0 | 0 | 0 | 0 | 0 | 0 | 0 | 0 | 0 | 0 | 0 | 13 | 0 |
| 6654e5e8b992b1433b5c2ed53317851a | *Sanguina* sp. DR74a_Trochiscia-type (OL962698) | 1-2 | 0 | 0 | 0 | 0 | 0 | 5.9 | 0 | 0 | 0 | 0 | 0 | 0 | 0 | 0 | 0 | 0 |
| 7abf0c736073c6ff8c89ec244b668536 | *Sanguina* sp. DR74a_Trochiscia-type (OL962698) | 1-2 | 0 | 0 | 0 | 0 | 0 | 0 | 0 | 21.8 | 0 | 0 | 0 | 0 | 0 | 0 | 0 | 0 |
| 8a6a8c3d2f06423d6ced2adcfeca2494 | *Sanguina* sp. DR74a_Trochiscia-type (OL962698) | 1-2 | 4 | 0 | 0 | 0 | 0 | 0 | 0 | 0 | 0 | 0 | 0 | 0 | 0 | 0 | 0 | 0 |
| a51eaba4ac579f6b8863f2ec7407d782 | *Raphidonema sempervirens* KMY-2018 SR1-B (MK262787) | 9 | 0 | 0 | 2.2 | 0 | 0.1 | 0.1 | 0 | 0 | 0 | 0 | 0 | 7.1 | 0 | 10.5 | 26.5 | 30.3 |
| aba9d873a6fb5654d98cbd44e93e727b | *Sanguina* sp. H14 (uncultured "*Chlamydomonas*" clone H14) (KX063729) | 1-2 | 0 | 0 | 0 | 0 | 0 | 0 | 0 | 0 | 0 | 0 | 0 | 4.7 | 87.7 | 26 | 25.8 | 0 |
| af293f8a780ee48706f3d933bd27de6a | *Sanguina* sp. H14 (uncultured "*Chlamydomonas*" clone H14) (KX063729) | 1-2 | 4 | 0 | 0 | 13 | 0 | 0 | 0 | 0 | 0 | 0 | 0 | 0 | 0 | 0 | 0 | 0 |
| c22f322dfe8ee0dbdea5834c2a34667c | *Sanguin*a sp. H14 (uncultured "*Chlamydomonas*" clone H14) (KX063729) | 1-2 | 0 | 0 | 0 | 0 | 0 | 0 | 0 | 13.9 | 0 | 0 | 0 | 0 | 0 | 0 | 0 | 0 |
| eff72301d5a69dee3cba4c6f40cce03f | *Sanguina aurantia* CCCryo RS_0017-2010 (MK728634) | 1-2 | 0 | 0 | 0 | 0 | 0 | 0 | 0 | 0 | 0 | 0 | 0 | 0 | 0 | 4.9 | 0 | 0 |
| fd0d87c7ef4dbdfb808224b65be0df42 | *Sanguina* sp. DR74a_Trochiscia-type (OL962698) | 1-2 | 83.8 | 0 | 0 | 0 | 0 | 0 | 0 | 0 | 0 | 0 | 0 | 0 | 0 | 0 | 0 | 0 |
| 81f99d3904b051b648e4cf8133cbeeef | *Raphidonema sempervirens* KMY-2018 SR1-B (MK262787) | 9 | 0 | 0 | 0 | 0 | 0 | 0 | 0 | 44.7 | 0 | 0 | 0 | 0 | 0 | 0 | 0 | 0 |
| 207ac8aa6085c6705b9fffbbb5f51095 | *Chloromonas* cf. *alpina* CCCryo 033-99 (HQ404865)^2^ | 7 | 0 | 0.7 | 0 | 0 | 0 | 0 | 0 | 0 | 0 | 0 | 0 | 0 | 0 | 0 | 0 | 0 |
| 647b244b03b2972806610d66b52f4a1c | *Chloromonas* sp. CCCryo 261-06 (HQ404889)^3^ | 7 | 0 | 0 | 2.4 | 60.6 | 0 | 0 | 0 | 0 | 0 | 0 | 0 | 0 | 0 | 0 | 0 | 0 |
| 650fd5d4fe6e3d7fc9a87212692002ce | *Chloromonas* sp. CCCryo 261-06 (HQ404889)^3^ | 7 | 0 | 0 | 0 | 0 | 0 | 1.4 | 0 | 16.3 | 0 | 0 | 0 | 0 | 0 | 0 | 0 | 0 |
| 8281dbf7b698436d5dcced2c69399082 | *Scotiella cryophila* K-1 (MG253843) | -- | 0 | 0 | 15.1 | 0 | 0 | 0 | 0 | 0 | 0 | 0 | 0 | 0 | 0 | 0 | 0 | 0 |
| 6f7df5825d3d3cd2e6183c6836026e49 | *Sanguina aurantia* CCCryo RS_0017-2010 (MK728634) | 1-2 | 0 | 0 | 0 | 0 | 0 | 0 | 37.7 | 0 | 0 | 0 | 0 | 0 | 0 | 0 | 0 | 0 |
| 7a4e6098e056a0b5a6dbc011ccf78070 | *Chloromonas* sp. CCCryo 261-06 (HQ404889)^3^ | 7 | 0 | 0 | 0 | 0 | 0 | 0 | 0 | 0 | 0 | 0 | 8.5 | 0 | 0 | 0 | 0 | 0 |
| 8527fe478f3d34aa468ebeb3d3202ec9 | no blast hit^4^ | 8 | 0 | 18 | 0 | 0 | 0 | 0 | 0 | 0 | 0 | 0 | 0 | 0 | 0 | 0 | 0 | 0 |
| c1daef59a4b085c6e161b90ca2008149 | *Koliellopsis inundata* FACHB-2451 (MT261819) | -- | 0 | 14 | 0 | 0 | 0 | 0 | 0 | 0 | 0 | 0 | 0 | 0 | 0 | 0 | 0 | 0 |
| 99b426bbd20fa941e62264791b538021 | no blast hit^5^ | 13 | 0 | 0 | 0 | 0 | 0 | 0 | 0 | 0 | 0 | 0 | 0 | 0 | 0 | 10.1 | 0 | 0 |
| 1f4dafe6e5ab25d464dde53c2413cd8c | *Trebouxia* sp. OTU S02 voucher UGDA:L-24319 (MN412818) | -- | 1.6 | 0 | 0 | 0 | 0 | 0 | 0 | 0 | 0 | 5.4 | 0 | 2 | 0 | 0 | 0 | 0 |
| 5c66985b0ad13744295c0c520829649d | no blast hit | -- | 0 | 0 | 0 | 20.5 | 0 | 0 | 0 | 0 | 0 | 0 | 0 | 0 | 0 | 0 | 0 | 0 |
| de1838381c97e2cf7228894d6ae9dab7 | no blast hit^4^ | 8 | 0 | 67.2 | 0 | 0 | 0 | 0 | 0 | 0 | 0 | 0 | 0 | 0 | 0 | 0 | 0 | 0 |
| 8e9e5aabd31825f9334d0bf5b5d79949 | no blast hit | -- | 0 | 0 | 23.7 | 0 | 0 | 0 | 0 | 0 | 0 | 0 | 0 | 0 | 0 | 0 | 0 | 0 |
| 638bf72122e3f4692b756dba9efb9b0d | no blast hit | -- | 0 | 0 | 0 | 0 | 0 | 0 | 0 | 0 | 0 | 0 | 0 | 0 | 0 | 0 | 0 | 36.4 |
| bf1be74c3533eb8d42d676433e647118 | no blast hit | -- | 0 | 0 | 0 | 0 | 0 | 0 | 0 | 0 | 0 | 74.9 | 15.4 | 0 | 0 | 0 | 0 | 0 |
| fc4204b726b2de3cb3345849b53aa845 | no blast hit | -- | 0 | 0 | 0 | 0 | 0 | 0 | 0 | 0.7 | 2.2 | 2.8 | 55 | 0 | 0 | 4 | 0 | 0 |
| e8635c546c979880c71fb0e84304fbaa | no blast hit^6^ | 12 | 0 | 0 | 0 | 0 | 0 | 0 | 0 | 0 | 16.8 | 0 | 0 | 0 | 0 | 0 | 0 | 0 |
| 1225d84c42c86fcb50f1cb3ebd20a30d | no blast hit | -- | 0 | 0 | 0 | 0 | 0 | 0 | 0 | 0 | 0 | 0 | 0 | 0 | 0 | 10.4 | 0 | 0 |
| f8da6d332f722ce86f2b9680074d9917 | *Chloromonas* sp. CCCryo 261-06 (HQ404889)^3^ | 7 | 0 | 0 | 0 | 0 | 0 | 0 | 0 | 2.6 | 0 | 0 | 0 | 7.6 | 0 | 0 | 0 | 5.9 |
| 1779883e6853dd16b4bdfe663f56fbdc | no blast hit^7^ | 11 | 0 | 0 | 0 | 0 | 0 | 0 | 0 | 0 | 0 | 0 | 0 | 0 | 0 | 0 | 34.7 | 0 |

^1^one CBC when compared to Uncultured alga OTU375 (LC381742)

^2^conspecific with *Chloromonas* sp. CCCryo 261-06 (HQ404889)

^3^conspecific with *Chloromonas* cf. *alpina* CCCryo 033-99 (HQ404865)

^4^one CBC compared to *Chlorominima collina* CCCryo 273-06 (HQ404890)

^5^only 85% identity compared to *Chlamydomonas proboscigera* CCCryo 2017-05 (GU117581)

^6^only 86% identity compared to *Ploeotila* sp. CCCryo 086-99 (HQ404867)

^7^three CBCs compared to Uncultured Chlorophyta clone ALBC6 (JX435348)

**Supplementary Table 5.** Cryoflora community structure based on the **ITS2** data set (amplified by the “ice“ primers for glacial algae), comprising the 51 most abundant ASVs. An identity threshold of ~89% against a reference had to be passed in order to be considered as a database match. Sequences below this threshold were recorded as “no blast hit”. In the next step, compensatory base change (CBC) in homologous base pair positions in ITS2 were identified as a proxy of species identification (done for the 21 most abundant algal ASVs, which contributed to >1% of reads at least in one of the samples). Unless ASVID was 100% identical with the reference, a CBC search was conducted and it was done in the entire ITS2 secondary structure (see **Supplementary Figures** **4-6 and 9** [column: SF]). Unless a CBC was found, the ASVID was assigned to the reference. If a CBC was found, the sequence was recorded as “no blast hit”. Moreover, haplotype network analyses applied on the 19 most abundant glacial algal ITS2 ASVs resulted in ten haplotypes of *Ancylonema nordenskioeldii* [H1-H10], five haplotypes of *Ancylonema alaskanum* [HA1-HA5] and a single haplotype of *Ancylonema* sp. [*; see the column: H].

| **ASV ID** | **Assignment** | **SF** | **H** | **WP**  **165** | **WP**  **166** | **WP**  **212** | **TAR**  **13-13** | **GrIS**  **16-10** | **GrIS**  **16-5** | **GrIS**  **16-9** | **SVA**  **13-18** | **SVA**  **13-50** |
| --- | --- | --- | --- | --- | --- | --- | --- | --- | --- | --- | --- | --- |
| 408c7fbb4195550995ecd2f1282ffed6 | *Ancylonema nordenskioeldii* WP211 (OL898470) | 6 | H1 | 0 | 0 | 34.68 | 10.40 | 85.79 | 42.38 | 94.49 | 26.76 | 45.86 |
| dd4e83a41c02b52efad4360be53e6177 | *Ancylonema alaskanum* WP167 (OL898466) | 5 | HA1 | 86.94 | 86.04 | 30.87 | 1.63 | 0.01 | 0 | 0 | 0.30 | 0.11 |
| 455e90e031ab0918cb4d186912312d4c | *Ancylonema nordenskioeldii* WP211 (OL898470) | 4, 6 | H2 | 0 | 0 | 8.74 | 66.71 | 3.35 | 0.14 | 0.06 | 17.06 | 16.32 |
| 307a662935a6385de734219e085fb029 | *Ancylonema nordenskioeldii* WP211 (OL898470) | 6 | H3 | 0 | 0 | 0 | 5.52 | 0.12 | 40.79 | 1.25 | 0 | 0 |
| 856dfae7c722ea762d5f9b58799ae002 | *Ancylonema nordenskioeldii* WP211 (OL898470) | 6 | H4 | 0 | 0 | 0 | 5.32 | 1.00 | 0.48 | 2.77 | 21.12 | 12.56 |
| bbf3ef85cd9add89ead2947300dfc447 | *Ancylonema* sp. | 4, 6 | * | 0.17 | 0.19 | 0.26 | 0.26 | 0 | 0 | 0 | 22.08 | 16.26 |
| c73f9c16b97974a210fe4989434e56e7 | *Ancylonema nordenskioeldii* WP211 (OL898470) | 6 | H5 | 2.36 | 2.66 | 12.16 | 0 | 0 | 0 | 0 | 0 | 0 |
| c0fc53b2d1e807a41d628ead97724270 | *Ancylonema alaskanum* WP167 (OL898466) | 5 | HA2 | 0.99 | 0.97 | 10.22 | 0.29 | 0 | 0 | 0 | 0.11 | 0 |
| ee8f268d5767c774c721a24ab618708a | *Ancylonema alaskanum* WP167 (OL898466) | 5 | HA3 | 5.04 | 6.27 | 0 | 0 | 0 | 0 | 0 | 0 | 0 |
| 11d4333aecb20d624c65cea245388b9f | *Ancylonema nordenskioeldii* WP211 (OL898470) | 6 | H6 | 0 | 0 | 0 | 0 | 0 | 10.01 | 0.48 | 0 | 0 |
| cd3ef42e2baa37dc919fb95aee4a1acc | *Ancylonema nordenskioeldii* WP211 (OL898470) | 4, 6 | H7 | 0 | 0 | 0 | 0 | 4.46 | 5.24 | 0.43 | 0 | 0 |
| 2e4770e44cf47cfb47924cb5c57c4311 | *Raphidonema sempervirens* KMY-2018 SR1-B (MK262787) | 9 |  | 0.23 | 0.41 | 0 | 0.43 | 0 | 0 | 0 | 3.46 | 3.40 |
| 2df72fd8c96e7e9e1e5e5563209fa758 | *Ancylonema nordenskioeldii* WP211 (OL898470) | 6 | H8 | 0 | 0 | 0 | 0.40 | 2.72 | 0 | 0.01 | 4.11 | 0 |
| a0d11f536af7761fb15c7175bbf870c9 | *Ancylonema alaskanum* WP167 (OL898466) | 5 | HA1 | 1.54 | 1.46 | 0 | 0 | 0 | 0 | 0 | 0 | 0 |
| 813fc42447027f9af697354e8728474d | *Ancylonema nordenskioeldii* WP211 (OL898470) | 6 | H9 | 0 | 0 | 0 | 2.92 | 0 | 0.07 | 0 | 0 | 0 |
| 08460e848c95505d0da010cee10c8cef | *Ancylonema nordenskioeldii* WP211 (OL898470) | 6 | H10 | 0 | 0 | 0 | 1.85 | 0 | 0 | 0 | 0.44 | 0.53 |
| c442cfd2ad8e8dfd0862ac1baacb29fa | *Raphidonema sempervirens* KMY-2018 SR1-B (MK262787) | 9 |  | 0 | 0 | 0 | 0 | 0 | 0 | 0 | 1.85 | 0.35 |
| ef0636dee5e1e85dd035471907cf8f1a | *Ancylonema alaskanum* WP167 (OL898466) | 5 | HA4 | 0 | 0 | 0 | 0.98 | 0 | 0 | 0 | 1.21 | 0 |
| c95c2e0cd30ddb5e51b84b648d53404a | *Ancylonema nordenskioeldii* WP211 (OL898470) |  |  | 0.91 | 0.90 | 0.31 | 0 | 0 | 0 | 0 | 0 | 0 |
| 95d818b6fa55141c9825ea90362b1173 | *Ancylonema nordenskioeldii* WP211 (OL898470) | 6 | H8 | 0 | 0 | 0 | 0 | 0 | 0 | 0 | 0 | 1.63 |
| 42d18b1bb1684377d22245873f651c36 | *Ancylonema alaskanum* WP167 (OL898466) | 5 | HA5 | 0 | 0 | 0 | 1.48 | 0 | 0 | 0 | 0 | 0 |
| e11dd3eacd2c6fc96a9373ae54ddabb5 | *Raphidonema sempervirens* KMY-2018 SR1-B (MK262787) |  |  | 0.18 | 0.23 | 0.84 | 0 | 0 | 0 | 0 | 0 | 0 |
| acb0c3c9c9e762c34df64f11159a88be | *Ancylonema alaskanum* WP167 (OL898466) |  |  | 0.83 | 0 | 0.28 | 0 | 0 | 0 | 0 | 0 | 0 |
| 656d3b9006e5cde90a1a494f7c1a8a8a | *Ancylonema nordenskioeldii* WP211 (OL898470) | 6 | H6 | 0 | 0 | 0 | 0 | 1.09 | 0 | 0 | 0 | 0 |
| aeefae679e4d2c96e8be5771fc8ce36f | *Ancylonema nordenskioeldii* WP211 (OL898470) |  |  | 0 | 0 | 0 | 0.58 | 0.16 | 0 | 0 | 0.16 | 0.18 |
| 0cece8f9735bc58baec091c043b97b36 | *Limnomonas svalbardensis* 217-05 (G117581) |  |  | 0 | 0 | 0 | 0 | 0 | 0 | 0 | 0 | 0.67 |
| c5e4abd842bbf7dfd2c02b9c8889c374 | *Ancylonema nordenskioeldii* WP211 (OL898470) |  |  | 0 | 0 | 0.08 | 0.04 | 0.17 | 0.08 | 0.21 | 0.01 | 0.02 |
| 0d09739893e2c94bea9d6366cd483e5a | *Ancylonema nordenskioeldii* WP211 (OL898470) |  |  | 0 | 0 | 0 | 0 | 0.49 | 0 | 0 | 0 | 0 |
| c274c9f2eded3732923ac59782a9ee30 | *Ancylonema nordenskioeldii* WP211 (OL898470) |  |  | 0.19 | 0.24 | 0.03 | 0 | 0 | 0 | 0 | 0 | 0 |
| a0986901dd7cd106711c2d8e76ce50fb | *Ancylonema nordenskioeldii* WP211 (OL898470) |  |  | 0 | 0 | 0.07 | 0.20 | 0 | 0 | 0 | 0.08 | 0.05 |
| e6c5fb16b2501294a766de6da59c2264 | No blast hit^1^ |  |  | 0 | 0 | 0 | 0 | 0 | 0.15 | 0 | 0 | 0.25 |
| 4112ade05ff707d15f2e199bdc7ad516 | *Ancylonema nordenskioeldii* WP211 (OL898470) |  |  | 0 | 0 | 0 | 0 | 0.40 | 0 | 0 | 0 | 0 |
| a15fff096c04c71223cfd8a3b9be11f4 | *Ancylonema nordenskioeldii* WP211 (OL898470) |  |  | 0 | 0 | 0.10 | 0 | 0.10 | 0.05 | 0 | 0.08 | 0.06 |
| da893671b9776cefb9fdb16293ae380c | *Ancylonema nordenskioeldii* WP211 (OL898470) |  |  | 0 | 0 | 0 | 0 | 0 | 0 | 0 | 0.12 | 0.24 |
| d02e285e7309686bbe35eacfa82176f8 | *Ancylonema nordenskioeldii* WP211 (OL898470) |  |  | 0 | 0 | 0.34 | 0 | 0 | 0 | 0 | 0 | 0 |
| 134b1c96325afade2f106ee137294932 | *Ancylonema alaskanum* WP167 (OL898466) |  |  | 0.14 | 0.12 | 0.06 | 0 | 0 | 0 | 0 | 0 | 0 |
| b9c84215f7e7f0271550283cc9b7c593 | *Ancylonema* sp. |  |  | 0 | 0 | 0 | 0 | 0 | 0 | 0 | 0.18 | 0.13 |
| f1a47344ac6e5e6f45c73b2250fa07fe | *Ancylonema alaskanum* WP167 (OL898466) |  |  | 0 | 0 | 0.29 | 0 | 0 | 0 | 0 | 0 | 0 |
| f80f865bc71c4d70a38eade6c5c606c3 | *Ancylonema nordenskioeldii* WP211 (OL898470) |  |  | 0 | 0 | 0 | 0 | 0 | 0.11 | 0 | 0.12 | 0.05 |
| c0e9a945962d572eabd88a894f3871c8 | *Ancylonema nordenskioeldii* WP211 (OL898470) |  |  | 0 | 0 | 0 | 0.27 | 0 | 0 | 0 | 0 | 0 |
| d84195a7df6dcdfdd483fcc0df3ba7be | *Ancylonema nordenskioeldii* WP211 (OL898470) |  |  | 0 | 0 | 0.22 | 0 | 0 | 0 | 0 | 0 | 0 |
| 4d5a146519bb364d625eed9df7f5e737 | *Ancylonema nordenskioldii* WP211 (OL898470) |  |  | 0.02 | 0.02 | 0.17 | 0 | 0 | 0 | 0 | 0 | 0 |
| faaa4b481b751f332c89e830f8e290a1 | *Ancylonema nordenskioeldii* WP211 (OL898470) |  |  | 0 | 0 | 0 | 0 | 0 | 0.21 | 0 | 0 | 0 |
| edddd4490f8d5a54f2f86ec0c043ca24 | *Ancylonema* sp. |  |  | 0 | 0 | 0 | 0 | 0 | 0 | 0 | 0.13 | 0.06 |
| 1f75c5b246695d563ddc8ed37de0601e | *Ancylonema nordenskioeldii* WP211 (OL898470) |  |  | 0 | 0 | 0 | 0 | 0 | 0.17 | 0 | 0 | 0 |
| f30215727789c584186a77ac282be5d1 | *Ancylonema alaskanum* WP167 (OL898466) |  |  | 0 | 0 | 0 | 0 | 0 | 0 | 0 | 0 | 0.17 |
| 0842b10340c3e936818d7fba759646dc | no blast hit^2^ |  |  | 0 | 0 | 0 | 0 | 0 | 0 | 0 | 0 | 0.16 |
| c42c5a86c1d1d3265cdfb51167f52e83 | *Ancylonema alaskanum* WP167 (OL898466) |  |  | 0 | 0 | 0 | 0.14 | 0 | 0 | 0 | 0 | 0 |
| 3842437e0670a2d873715a0c86d206f6 | *Ancylonema nordenskioeldii* WP211 (OL898470) |  |  | 0 | 0 | 0 | 0 | 0 | 0 | 0 | 0.06 | 0.08 |
| 535d4eab8501810e9f448b45b04d1ec3 | *Raphidonema sempervirens* KMY-2018 SR1-B (MK262787) |  |  | 0 | 0 | 0 | 0.06 | 0 | 0 | 0 | 0 | 0.08 |
| 135be8a3624b792c2746d21651db3700 | *Ancylonema nordenskioeldii* WP211 (OL898470) |  |  | 0 | 0 | 0 | 0 | 0 | 0 | 0.13 | 0 | 0 |
| other (less abundant ASVs) |  |  |  | 0.27 | 0.25 | 0.20 | 0.53 | 0.14 | 0.12 | 0.17 | 0.25 | 0.39 |

^1^<90% identity with *Cylindrocystis cushleckae* isolate CCAC 0213 (MN585756)

^2^only 83% identity with *Limnomonas svalbardensis* strain CCCryo 217-05 (G117581)

**Supplementary Table 6:** Amount of compensatory base changes in the ITS2 secondary structure among the most abundant ASV IDs assigned by blast search to *Ancylonema nordenskioeldii* WP211 (OL898470), and when compared with the reference sequence.

|  | *Ancylonema nordenskioldii*_WP211 | 656d3b9006e5cde90a1a494f7c1a8a8a | 813fc42447027f9af697354e8728474d | 95d818b6fa55141c9825ea90362b1173 | 408c7fbb4195550995ecd2f1282ffed6 | 455e90e031ab0918cb4d186912312d4c | 307a662935a6385de734219e085fb029 | 856dfae7c722ea762d5f9b58799ae002 | bbf3ef85cd9add89ead2947300dfc447 | c73f9c16b97974a210fe4989434e56e7 | 11d4333aecb20d624c65cea245388b9f | cd3ef42e2baa37dc919fb95aee4a1acc | 2df72fd8c96e7e9e1e5e5563209fa758 | 08460e848c95505d0da010cee10c8cef |
| --- | --- | --- | --- | --- | --- | --- | --- | --- | --- | --- | --- | --- | --- | --- |
| *Ancylonema nordenskioldii*_WP211 |  | 0 | 0 | 0 | 0 | 0 | 0 | 0 | **2** | 0 | 0 | 0 | 0 | 0 |
| 656d3b9006e5cde90a1a494f7c1a8a8a | 0 |  | 0 | 0 | 0 | 0 | 0 | 0 | **2** | 0 | 0 | 0 | 0 | 0 |
| 813fc42447027f9af697354e8728474d | 0 | 0 |  | 0 | 0 | 0 | 0 | 0 | **2** | 0 | 0 | 0 | 0 | 0 |
| 95d818b6fa55141c9825ea90362b1173 | 0 | 0 | 0 |  | 0 | 0 | 0 | 0 | **2** | 0 | 0 | 0 | 0 | 0 |
| 408c7fbb4195550995ecd2f1282ffed6 | 0 | 0 | 0 | 0 |  | 0 | 0 | 0 | **2** | 0 | 0 | 0 | 0 | 0 |
| 455e90e031ab0918cb4d186912312d4c | 0 | 0 | 0 | 0 | 0 |  | 0 | 0 | **2** | 0 | 0 | 0 | 0 | 0 |
| 307a662935a6385de734219e085fb029 | 0 | 0 | 0 | 0 | 0 | 0 |  | 0 | **2** | 0 | 0 | 0 | 0 | 0 |
| 856dfae7c722ea762d5f9b58799ae002 | 0 | 0 | 0 | 0 | 0 | 0 | 0 |  | **2** | 0 | 0 | 0 | 0 | 0 |
| bbf3ef85cd9add89ead2947300dfc447 | **2** | **2** | **2** | **2** | **2** | **2** | **2** | **2** |  | **2** | **2** | **3** | **2** | **2** |
| c73f9c16b97974a210fe4989434e56e7 | 0 | 0 | 0 | 0 | 0 | 0 | 0 | 0 | **2** |  | 0 | 0 | 0 | 0 |
| 11d4333aecb20d624c65cea245388b9f | 0 | 0 | 0 | 0 | 0 | 0 | 0 | 0 | **2** | 0 |  | 0 | 0 | 0 |
| cd3ef42e2baa37dc919fb95aee4a1acc | 0 | 0 | 0 | 0 | 0 | 0 | 0 | 0 | **3** | 0 | 0 |  | 0 | 0 |
| 2df72fd8c96e7e9e1e5e5563209fa758 | 0 | 0 | 0 | 0 | 0 | 0 | 0 | 0 | **2** | 0 | 0 | 0 |  | 0 |
| 08460e848c95505d0da010cee10c8cef | 0 | 0 | 0 | 0 | 0 | 0 | 0 | 0 | **2** | 0 | 0 | 0 | 0 |  |


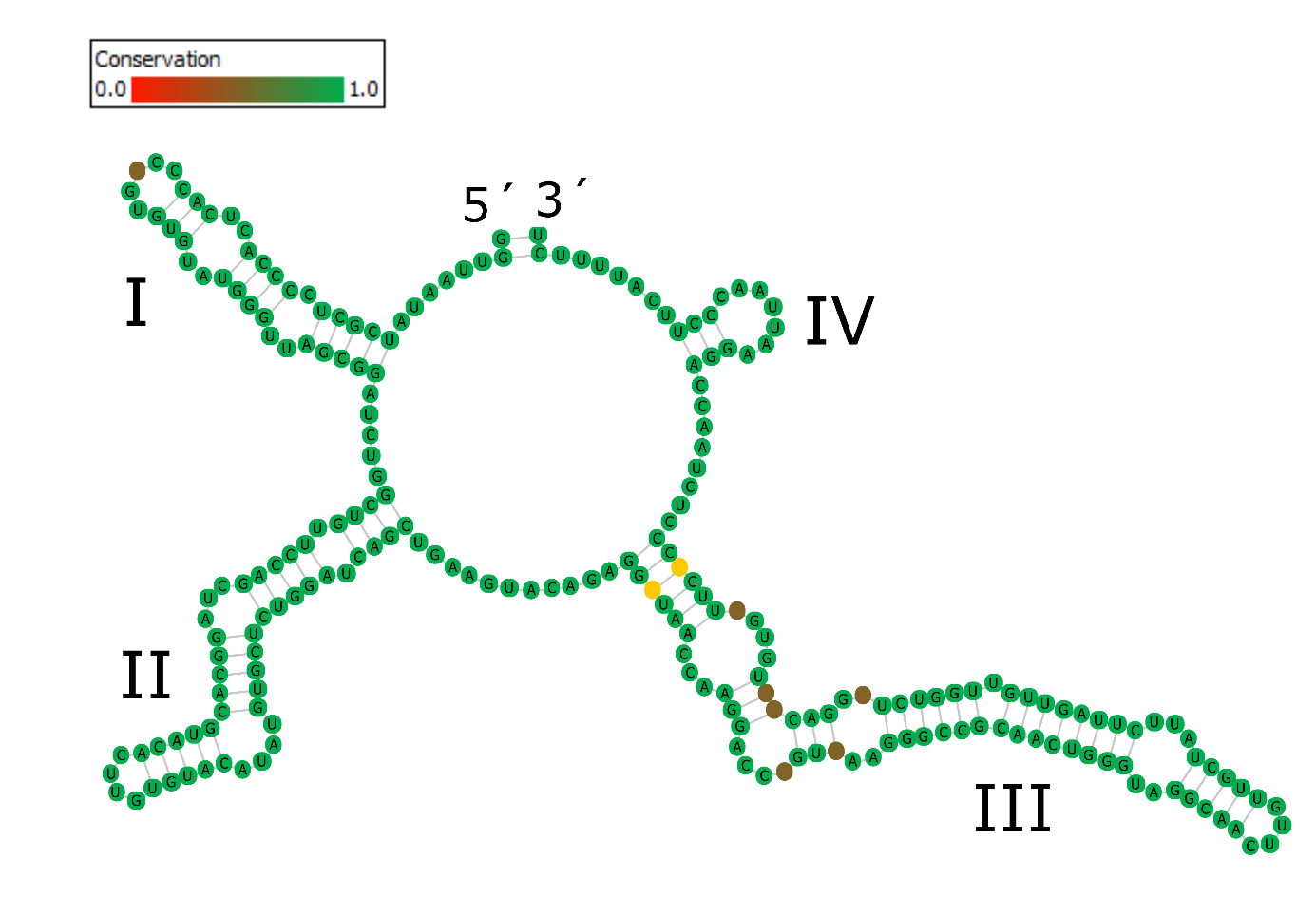


**Supplementary Figure 1.** Sequence conservation of nuclear ribosomal DNA (rRNA gene ) internal transcribed spacer 2 (ITS2) among two out of the most abundant ASV IDs in this study (>1% of reads per any of the samples; 8527fe478f3d34aa468ebeb3d3202ec9 and de1838381c97e2cf7228894d6ae9dab7) and their reference sequences these ASV IDs were assigned by blast search: *Chlorominima collina* CCCryo 273-06 (HQ404890), uncultured alga OTU101 (Stephanosphaerinia, LC381738). The structure of the nuclear rRNA gene ITS2 shows the common four helices. Sequence conservation is visualised via colour interpolation between red (not conserved) and green (conserved). No CBC was found between the two references. No CBC was found between the two ASV IDs. As contrast, **one CBC** was found in helix III between the ASV IDs and the reference sequences (highlighted in yellow). The consensus structure was generated using 4SALE (Seibel et al. 2006, 2008).


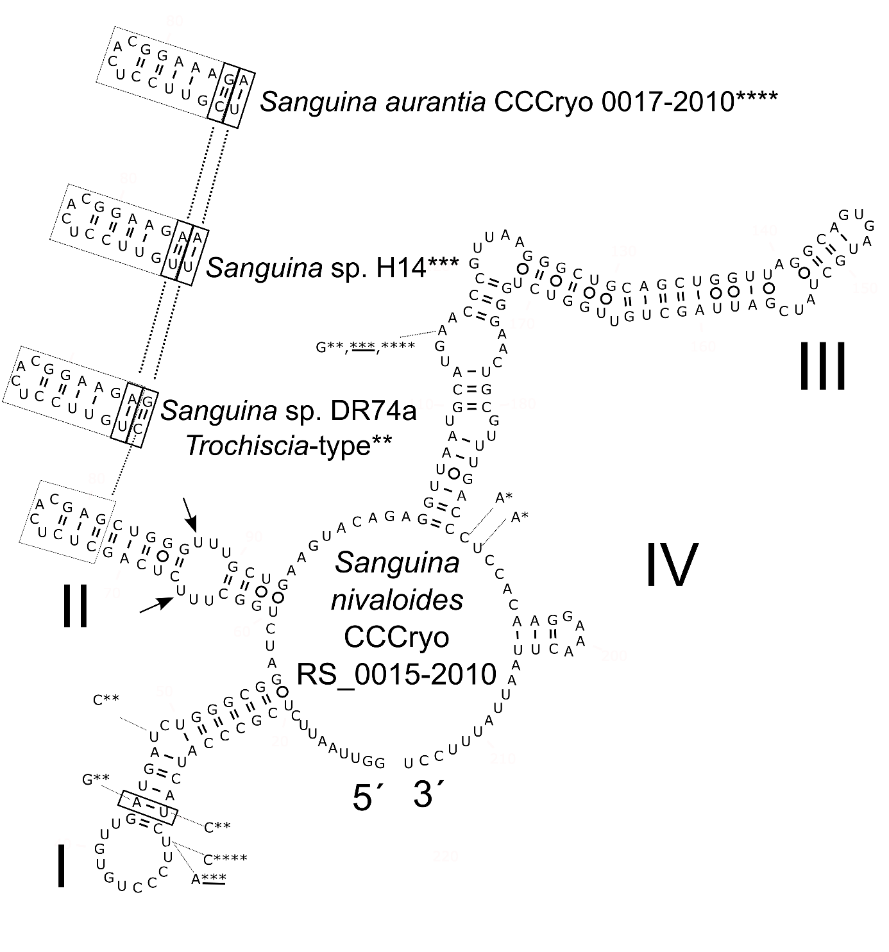


**Supplementary Figure 2**: Comparison of the secondary structure of ITS2 transcripts between four *Sanguina* species. Differences in the helix II between *Sanguina nivaloides* (the type specimen of CCCryo RS 0015-2010, MK728599.1; identical with ASV ID:6e1818ae977266295a32e31102b53d80 except from two bases [highlighted by a single asterisk]), *Sanguina* sp. DR74a *Trochiscia*-type (the type specimen OL962698; identical with ASV ID: fd0d87c7ef4dbdfb808224b65be0df42), *Sanguina* sp. H14 (uncultured clone H14, KX063729; identical with ASV ID: af293f8a780ee48706f3d933bd27de6a; except from two bases [highlighted by three underlined asterisks]) and *Sanguina aurantia* (the type specimen CCCryo0017-2010, MK728634; identical with ASVID:399608d04f0c5a06160d54d657ac40fe) are shown by characters just outside the secondary structure. Double asterisk means that it was found *Sanguina* sp. DR74a *Trochiscia*-type, triple asterisks (not underlined) indicates the presence in *Sanguina* sp. H14 and four asterisks shows that such nucleotide difference was found for *Sanguina aurantia* CCCryo0017-2010. The position of the compensatory base changes are indicated by rectangles. Note the U-U mismatch in helix II (arrowheads).


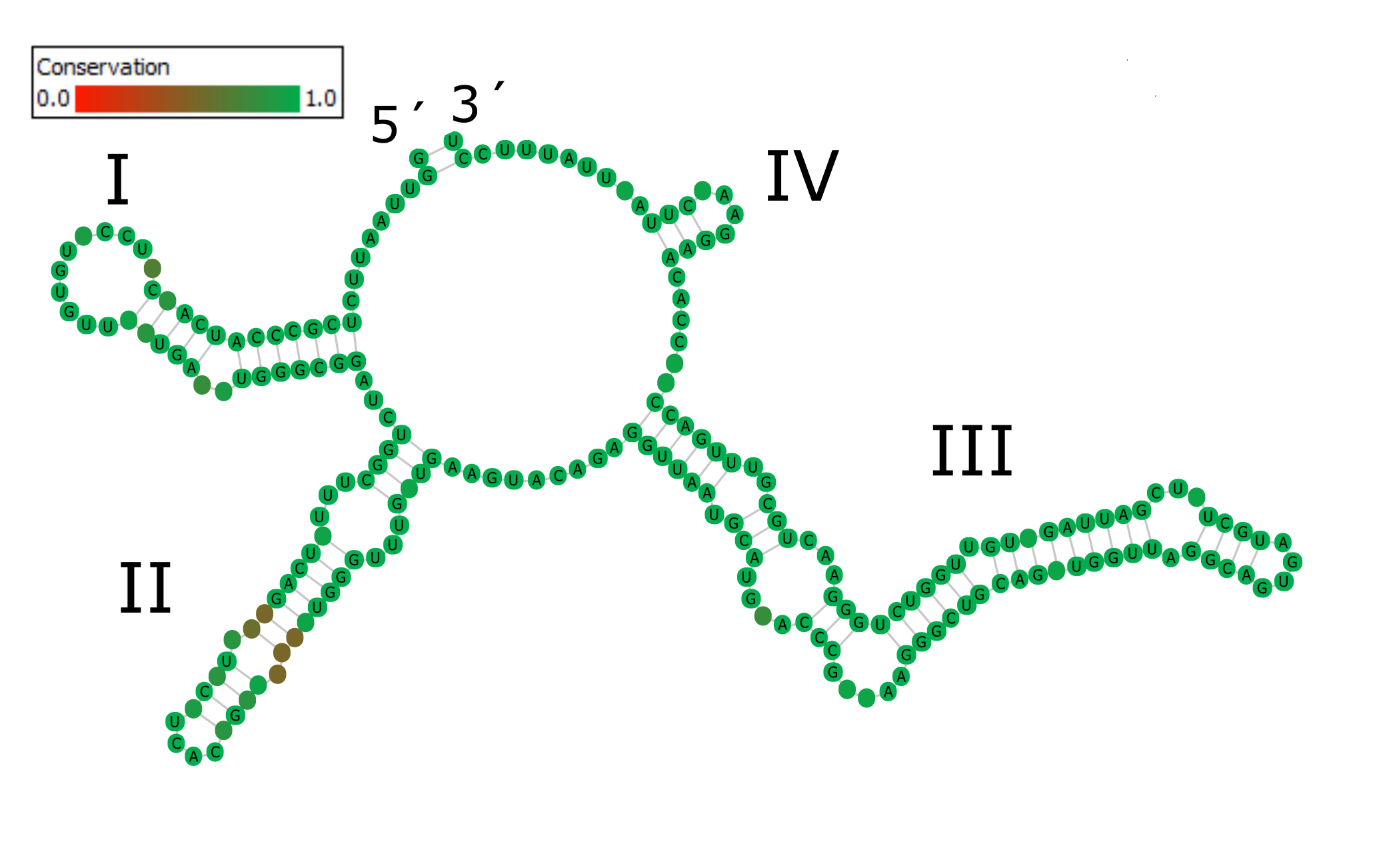


**Supplementary Figure 3.** Sequence conservation of nuclear ribosomal DNA (rRNA gene ) internal transcribed spacer 2 (ITS2) among the 15 out of the most abundant ASV IDs in this study (>1% of reads per any of the samples): *Sanguina nivaloides* (one ASV ID: 6e1818ae977266295a32e31102b53d80), *Sanguina* sp. DR74a *Trochiscia*-type (six ASV IDs: fd0d87c7ef4dbdfb808224b65be0df42, 20ce236c0ec95982dcb59eafed4bf026, 4985407c121d5195bcb5685fbb9e2e30, 6654e5e8b992b1433b5c2ed53317851a, 7abf0c736073c6ff8c89ec244b668536, 8a6a8c3d2f06423d6ced2adcfeca2494), *Sanguina* sp. H14 (three ASV IDs: aba9d873a6fb5654d98cbd44e93e727b, af293f8a780ee48706f3d933bd27de6a, c22f322dfe8ee0dbdea5834c2a34667c) and *Sanguina aurantia* (five ASV IDs: 6f7df5825d3d3cd2e6183c6836026e49, 399608d04f0c5a06160d54d657ac40fe, 505d0a0b136693647e331821956c14d3, 586563f91f085f301a20dd6e8051db61, eff72301d5a69dee3cba4c6f40cce03f). The structure of the nuclear rRNA gene ITS2 among the four species shows the common four helices. Sequence conservation is visualised via colour interpolation between red (not conserved) and green (conserved). The least conserved region in helix II indicates positions of CBC(s). The consensus structure was generated using 4SALE (Seibel et al. 2006, 2008).


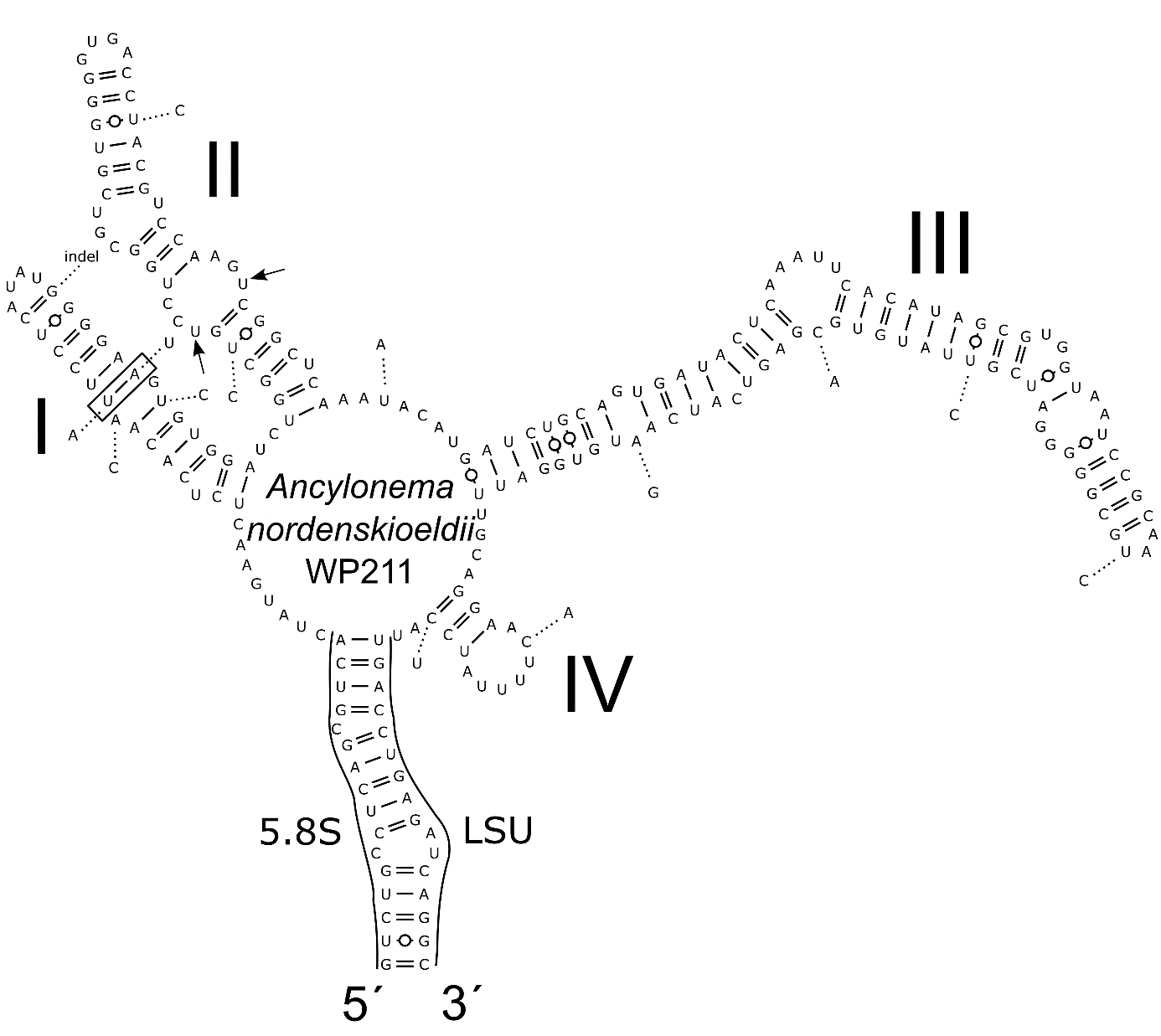


**Supplementary Figure 4**: Comparison of the secondary structure of ITS2 transcripts between *Ancylonema nordenskioeldii* (WP211; OL898470) and *Ancylonema alaskanum* (WP167; OL898466), including the 3’ end of the 5.8S ribosomal RNA (rRNA) and the 5’ end of the LSU rRNA. Note the U-U mismatch in helix II (arrowheads). Differences are shown by characters just outside the secondary structure, a box indicates a compensatory base change between these two species.


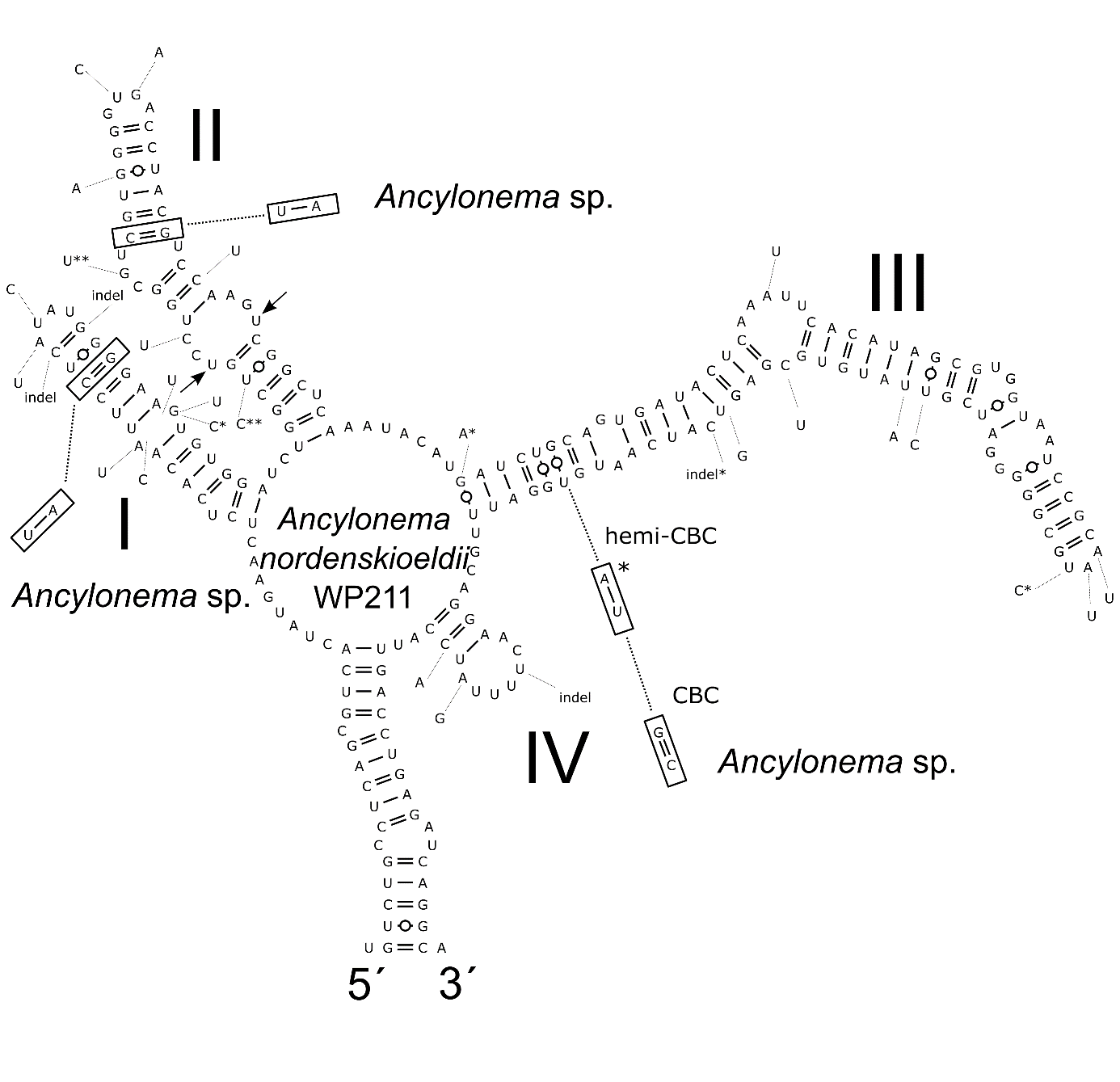


**Supplementary Figure 5**: Comparison of the secondary structure of ITS2 transcripts between the *Ancylonema nordenskioeldii* (ASV ID 455e90e031ab0918cb4d186912312d4c – identical with the reference sequence WP211 - OL898470; cd3ef42e2baa37dc919fb95aee4a1acc) and putative *Ancylonema* sp. (ASV ID bbf3ef85cd9add89ead2947300dfc447). Differences are shown by characters just outside the secondary structure, no asterisk means that the difference was found only in putative *Ancylonema* sp. ASV ID bbf3ef85cd9add89ead2947300dfc447, one asterisk implies that the change was in *Ancylonema nordenskioeldii* ASV ID cd3ef42e2baa37dc919fb95aee4a1acc, double asterisk indicates that the change was found in the both ASV IDs. The positions of the compensatory base change are indicated by rectangles, one hemi-CBC is indicated as well. Note the U-U mismatch in helix II (arrowheads).


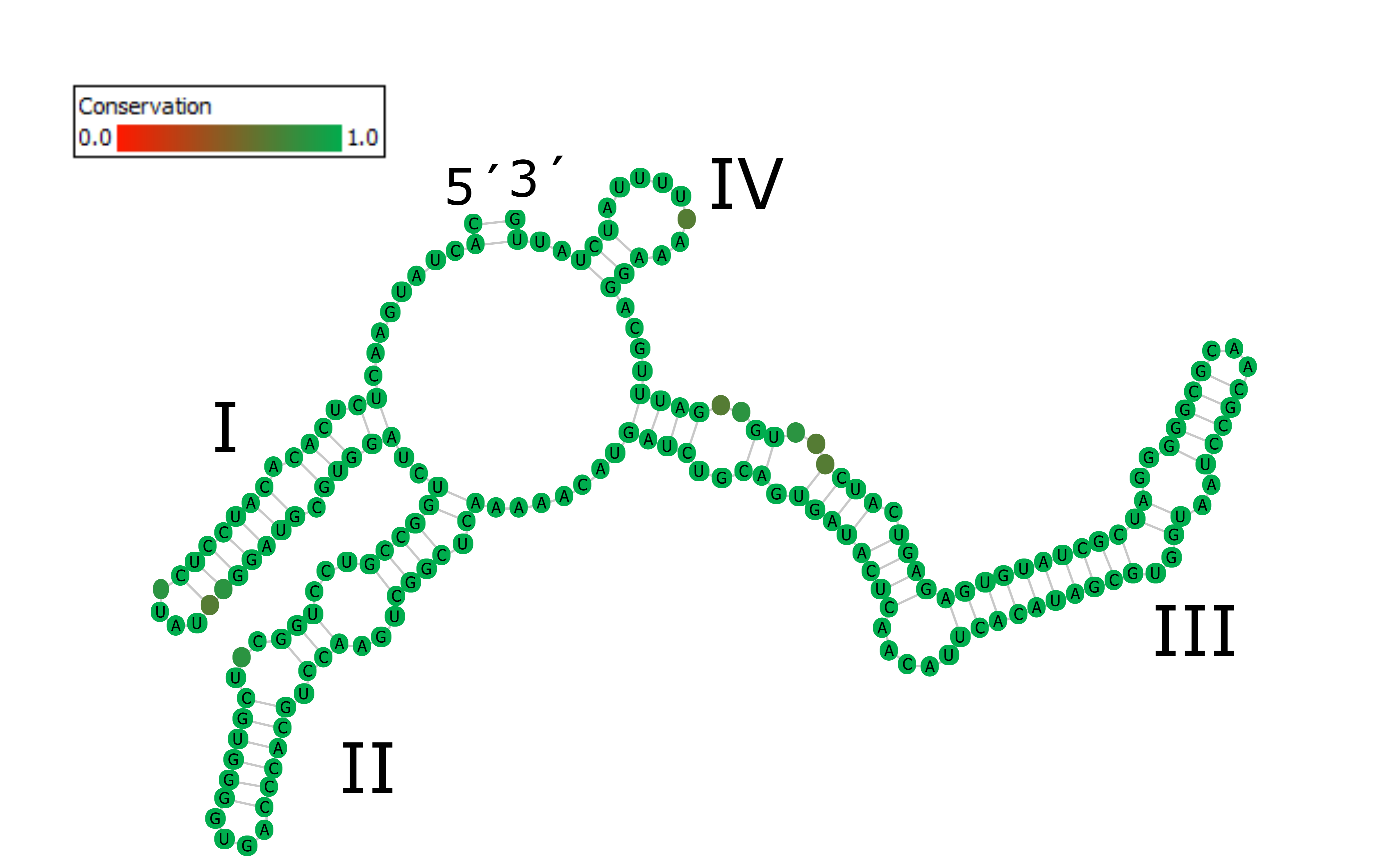


**Supplementary Figure 6.** Sequence conservation of nuclear ribosomal DNA (rRNA gene ) internal transcribed spacer 2 (ITS2) among the six out of the most abundant ASV IDs in this study (>1% of reads per any of the samples): assigned by blast search to *Ancylonema alaskanum* WP167 (OL898466). ASV IDs included in this graph: dd4e83a41c02b52efad4360be53e6177, c0fc53b2d1e807a41d628ead97724270, ee8f268d5767c774c721a24ab618708a, a0d11f536af7761fb15c7175bbf870c9, ef0636dee5e1e85dd035471907cf8f1a and 42d18b1bb1684377d22245873f651c36. The structure of the nuclear rRNA gene ITS2 shows the common four helices. Sequence conservation is visualised via colour interpolation between red (not conserved) and green (conserved). **No CBC** was found. The consensus structure was generated using 4SALE (Seibel et al. 2006, 2008).

**
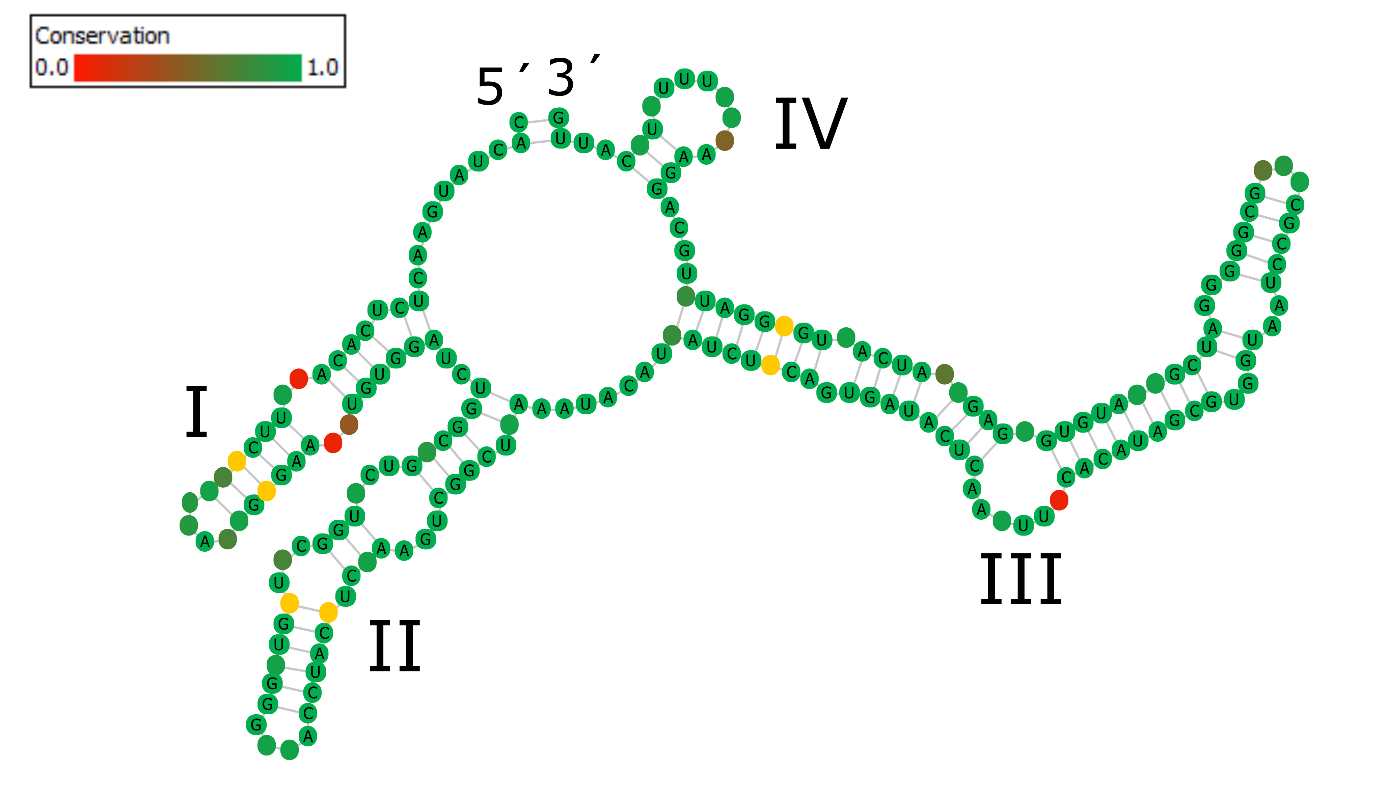
**

**Supplementary Figure 7.** Sequence conservation of nuclear ribosomal DNA (rRNA gene) internal transcribed spacer 2 (ITS2) among the 13 out of the most abundant ASV IDs in this study (>1% of reads per any of the samples) and the reference sequence these ASV IDs were assigned by blast search to: *Ancylonema nordenskioeldii* WP211 (OL898470). ASV IDs included in this graph are shown in **Supplementary** **Table 6**. The structure of the nuclear rRNA gene ITS2 shows the common four helices. Sequence conservation is visualised via colour interpolation between red (not conserved) and green (conserved). Except from ASV ID bbf3ef85cd9add89ead2947300dfc447, no CBC was found between ASV IDs and the reference sequence. As contrast, **two or even three CBCs** (highlighted in yellow) were detected between the ASV ID bbf3ef85cd9add89ead2947300dfc447 and all other sequences in the dataset (see **Supplementary Table 9**), consequently the former was assigned to *Ancylonema* sp. The consensus structure was generated using 4SALE (Seibel et al. 2006, 2008).


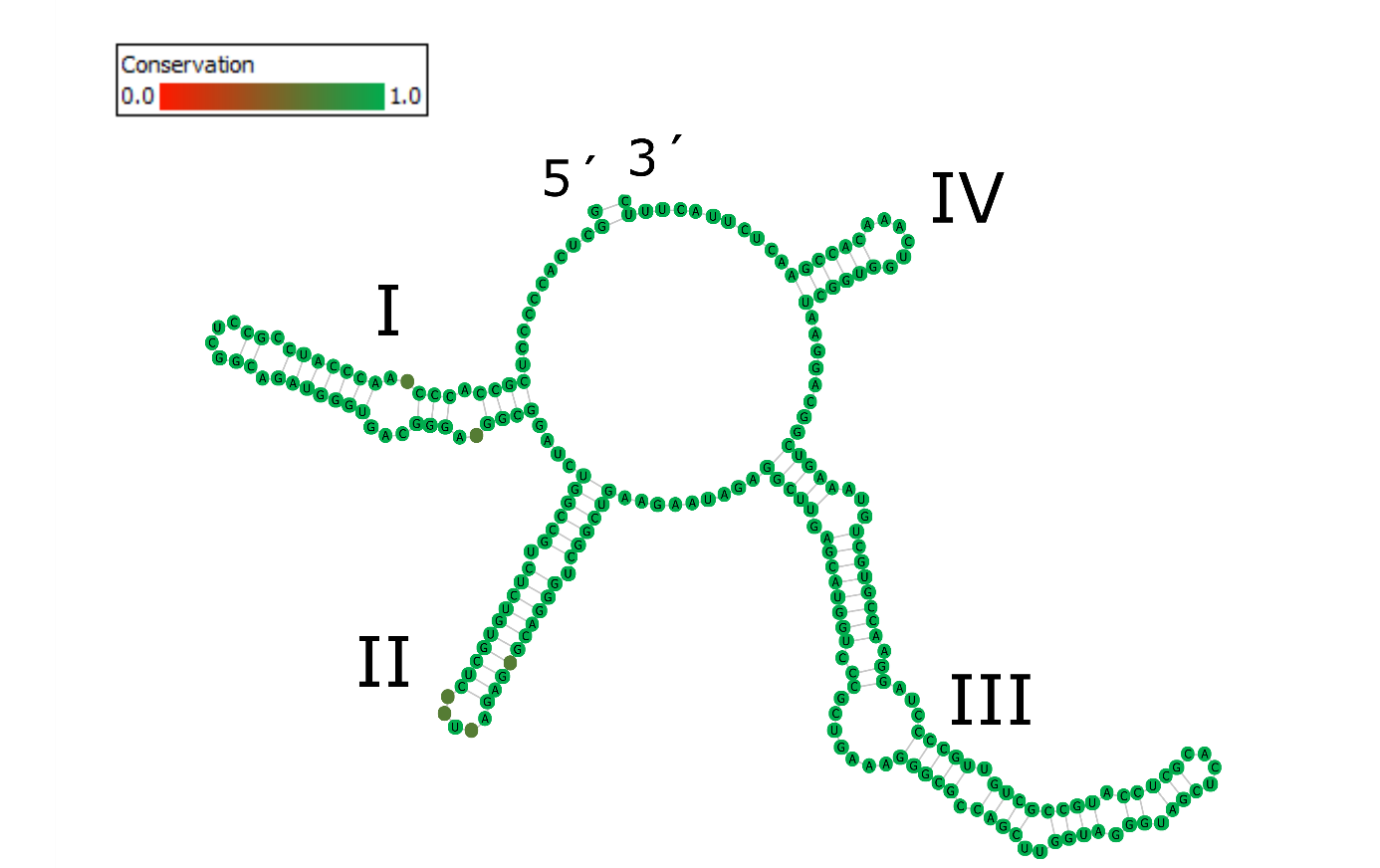


**Supplementary Figure 8.** Sequence conservation of nuclear ribosomal DNA (rRNA gene) internal transcribed spacer 2 (ITS2) among four out of the most abundant ASV IDs in this study (>1% of reads per any of the samples; HTS output with the primers for snow algae: a51eaba4ac579f6b8863f2ec7407d782, 81f99d3904b051b648e4cf8133cbeeef; HTS output with the primers designed for glacial algae: c442cfd2ad8e8dfd0862ac1baacb29fa, 2e4770e44cf47cfb47924cb5c57c4311) and the reference sequences these ASV IDs were assigned to by blast search: *Raphidonema sempervirens* KMY-2018 SR1-B (MK262787). The structure of the nuclear rRNA gene ITS2 shows the common four helices. Sequence conservation is visualised via colour interpolation between red (not conserved) and green (conserved). **No CBC** was found. The consensus structure was generated using 4SALE (Seibel et al. 2006, 2008).


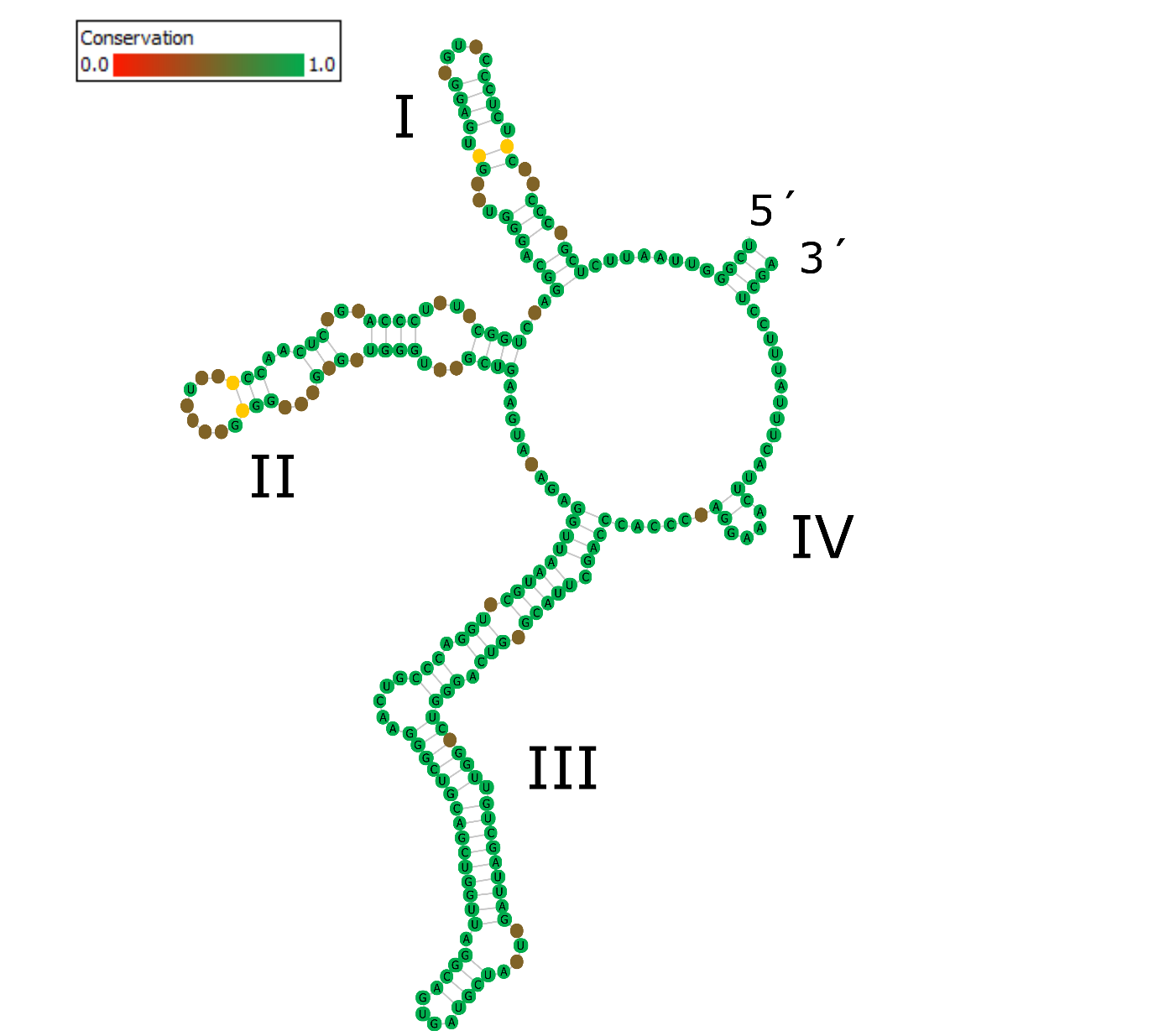


**Supplementary Figure 9.** Sequence conservation of nuclear ribosomal DNA (rRNA gene) internal transcribed spacer 2 (ITS2) among one of the most abundant ASV IDs in this study (>1% of reads per any of the samples; e8635c546c979880c71fb0e84304fbaa) and the reference sequence this ASV ID was assigned to by blast search: *Ploeotila* sp. CCCryo086-99 (HQ404867). The structure of the nuclear rRNA gene ITS2 shows the common four helices. Sequence conservation is visualised via colour interpolation between red (not conserved) and green (conserved). No CBC in the conserved part of the ITS2 structure; two CBCs were found in the expansion parts of the structure at the end of apex II and in close to the apex in helix I) when the ASV ID was compared with the reference sequence. The consensus structure was generated using 4SALE (Seibel et al. 2006, 2008).


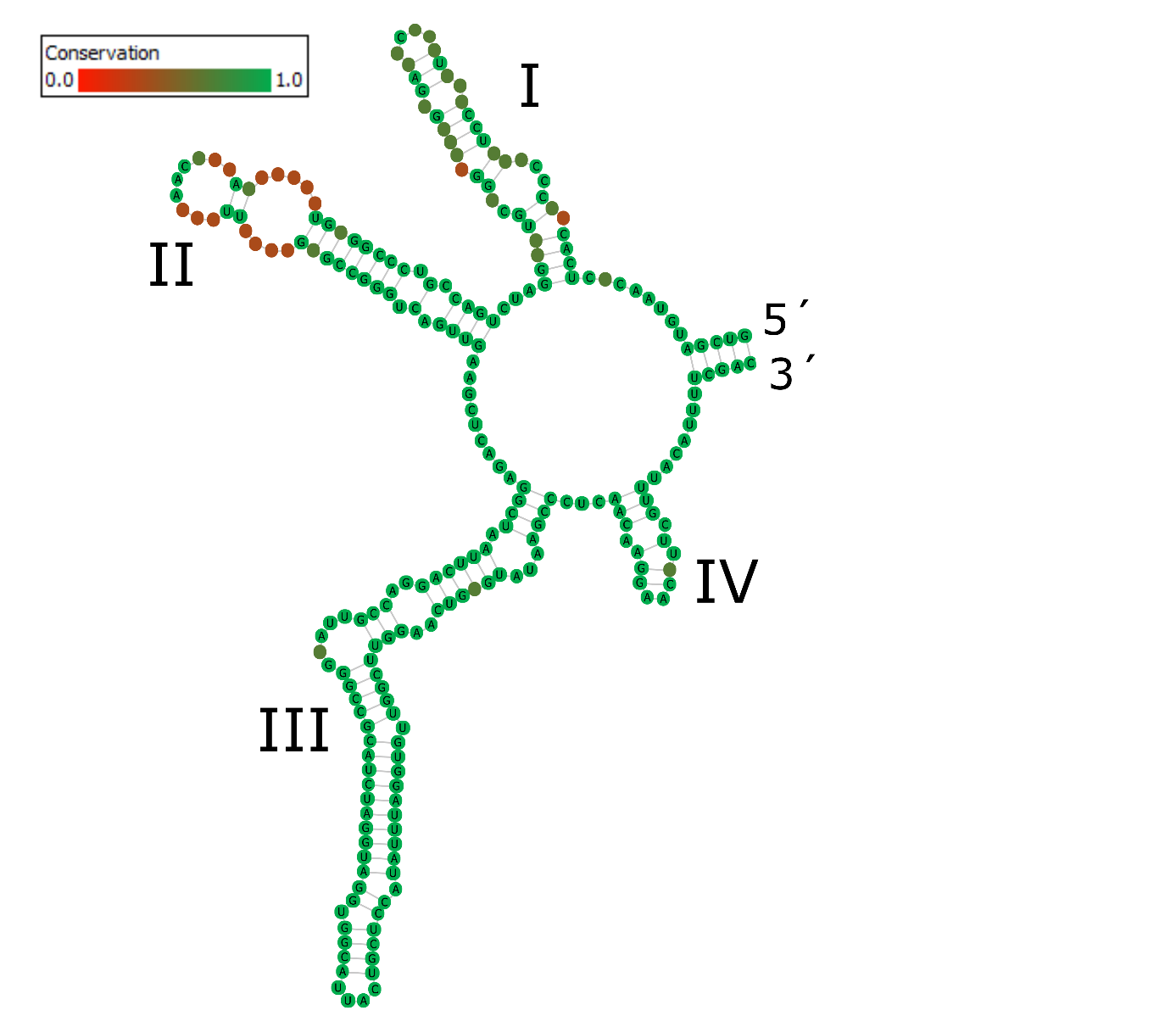


**Supplementary Figure 10.** Sequence conservation of nuclear ribosomal DNA (rRNA gene) internal transcribed spacer 2 (ITS2) among one of the most abundant ASV IDs in this study (>1% of reads per any of the samples; 99b426bbd20fa941e62264791b538021 and 28f2b3f21bed71b8dc2196a80189ff33) and the reference sequence this ASV ID was assigned to by blast search: *Limnomonas svalbardensis* CCCryo 217-05 (GU117581). The structure of the nuclear rRNA gene ITS2 shows the common four helices. Sequence conservation is visualised via colour interpolation between red (not conserved) and green (conserved). **No CBC** was found when the ASV ID was compared with the reference sequence. The consensus structure was generated using 4SALE (Seibel et al. 2006, 2008).


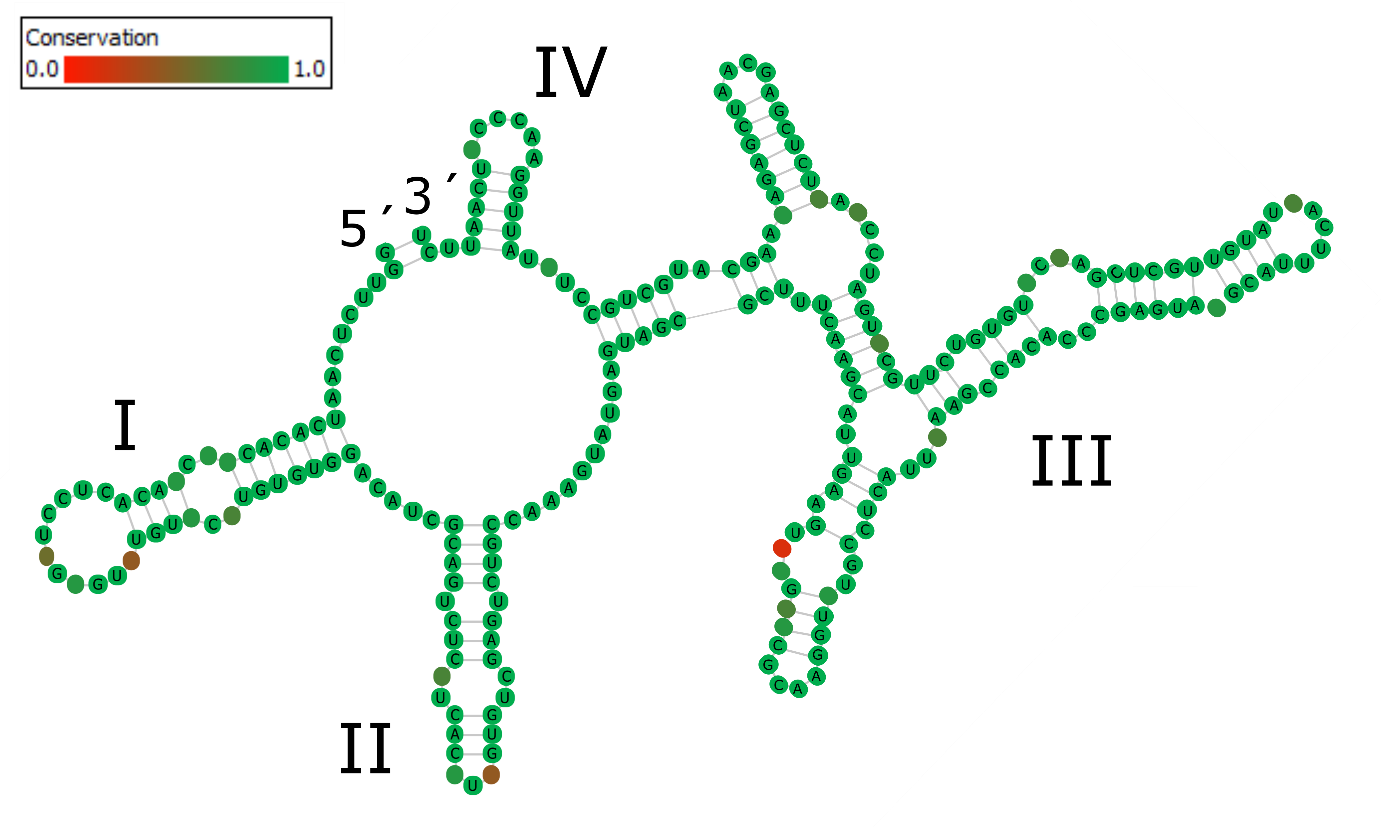


**Supplementary Figure 11.** Sequence conservation of nuclear ribosomal DNA (rRNA gene) internal transcribed spacer 2 (ITS2) among six out of the most abundant ASV IDs in this study (>1% of reads per any of the samples), and the reference sequences of these ASVIDs were assigned by blast search: *Chloromonas* cf. *alpina* CCCryo 033-99 (HQ404865; one ASV ID in this study: 207ac8aa6085c6705b9fffbbb5f51095) and *Chloromonas* sp. CCCryo 261-06 (HQ404889; five ASV IDs in this study: 647b244b03b2972806610d66b52f4a1c, 650fd5d4fe6e3d7fc9a87212692002ce, ee3b421e1e2dcf8c02c4a0cffb6763d5, 7a4e6098e056a0b5a6dbc011ccf78070, f8da6d332f722ce86f2b9680074d9917). The structure of the nuclear rRNA gene ITS2 shows the common four helices. Sequence conservation is visualised via colour interpolation between red (not conserved) and green (conserved). **No CBC** was found. The consensus structure was generated using 4SALE (Seibel et al. 2006, 2008).


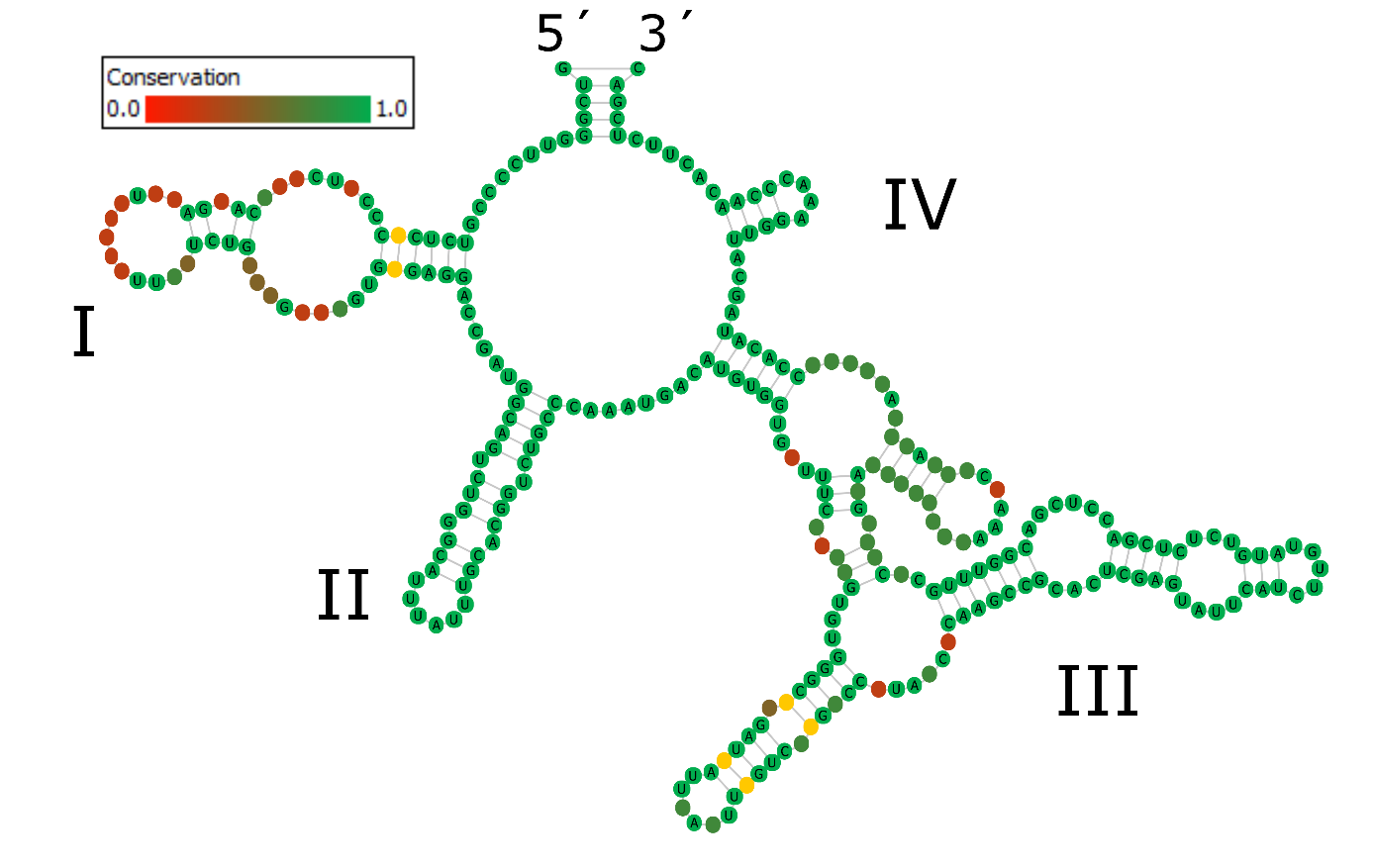


**Supplementary Figure 12.** Sequence conservation of nuclear ribosomal DNA (rRNA gene) internal transcribed spacer 2 (ITS2) among two out of the most abundant ASV IDs in this study (>1% of reads per any of the samples; befe347a0eb994f55693cc01c6f0bc90 and 1779883e6853dd16b4bdfe663f56fbdc) and the reference sequences these ASV IDs were assigned to by blast search: Uncultured Chlorophyta clone ALBC6 (JX435348). The structure of the nuclear rRNA gene ITS2 shows the common four helices. Sequence conservation is visualised via colour interpolation between red (not conserved) and green (conserved). While ASV ID befe347a0eb994f55693cc01c6f0bc90 is identical with the reference sequence, for 1779883e6853dd16b4bdfe663f56fbdc - **one CBC** was found in helix I (yellow) and **two CBCs** in the most conserved helix III when compared with the reference sequence. The consensus structure was generated using 4SALE (Seibel et al. 2006, 2008).


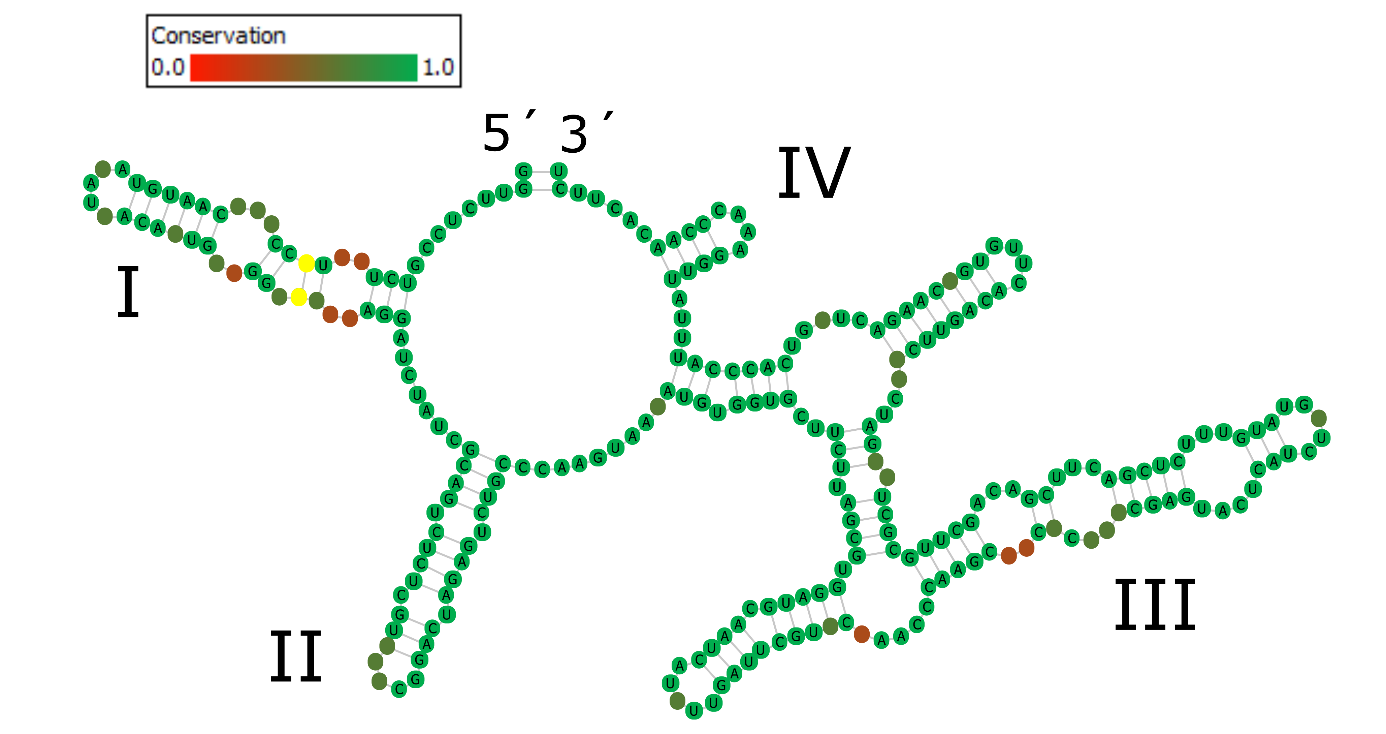


**Supplementary Figure 13.** Sequence conservation of nuclear ribosomal DNA (rRNA gene) internal transcribed spacer 2 (ITS2) among two out of the most abundant ASV IDs in this study (>1% of reads per any of the samples; 81f5ec709b7448ed342d146c70fe8f49 and 8a2f13dbcdfda0f84aae43485a0f8829) and the reference sequences these ASV IDs were assigned to by blast search: uncultured alga OTU375 (LC381742; Chloromonadinia group A in Segawa et al. 2018). The structure of the nuclear rRNA gene ITS2 shows the common four helices. Sequence conservation is visualised via colour interpolation between red (not conserved) and green (conserved). **One CBC** was found in helix I (yellow) when compared with the reference sequence. The consensus structure was generated using 4SALE (Seibel et al. 2006, 2008).

**Prokaryotic Community Structure**

Generation and processing of 16S rRNA sequences

16S rRNA genes were amplified using the bacterial primers 341F (5’-CCTACGGGNGGCWGCAG) and 785R (5’-GACTACHVGGGTATCTAATCC) spanning the V3-V4 hypervariable regions. 16S paired-end reads were quality filtered, trimmed and denoised into amplicon sequence variants (ASV) using dada2. The first 10 bp of each read were trimmed off. Forward reads were truncated at 280 bp and reverse reads at 250 bp in order to remove low quality regions. ASVs were annotated using a Naive Bayes classifier pre-trained on the full length Greengenes (v.13.8) database. Sequences matching plastidal DNA (i.e., Chloroplast, Mitochondria) were removed from the 16S dataset. The feature table was rarified using the lowest common number of sequences per sample (i.e., 7100) and only ASVs with a minimum frequency of 10 across all samples were retained.

Results and Discussion

Bacteria clustered according to habitats (R=0.6625, p=0.001) with Betaproteobacteria and Bacteriodetes being more abundant in snow and Alphaproteobacteria, Cyanobacteria, Acidobacteria and Actinobacteria being more abundant on ice. Overall, heterotrophic bacteria dominated in all samples. Phototrophic cyanobacteria (Synechococcophycidae) were only abundant on glaciers in Greenland, Svalbard and the Alps, but not in snow substrate. The full community composition can be found in Supplementary Table 2.

Cyanobacteria (mainly *Leptolyngbya* and *Pseudanabaena*) were abundantly found at the studied glaciers. Certainly, such prokaryotes are dominating in cryoconite holes on glacier surfaces (Segawa et al. 2017). Due to the transition between dirty ice and such holes, there is a large community similarity between these two ecosystems within one locus (Lutz et al. 2016). In contrast, the absence of cyanobacteria in the studied snow samples is consistent with earlier reports (Quesada and Vincent 2012, Cepák et al. 2016) and may reflect low growth rates, therefore not able to mitigate continuous population losses e.g. due to percolating meltwater. Scarce reports of cyanobacteria in snow were usual at glacier-based sites (Takeuchi et al. 2009).

**
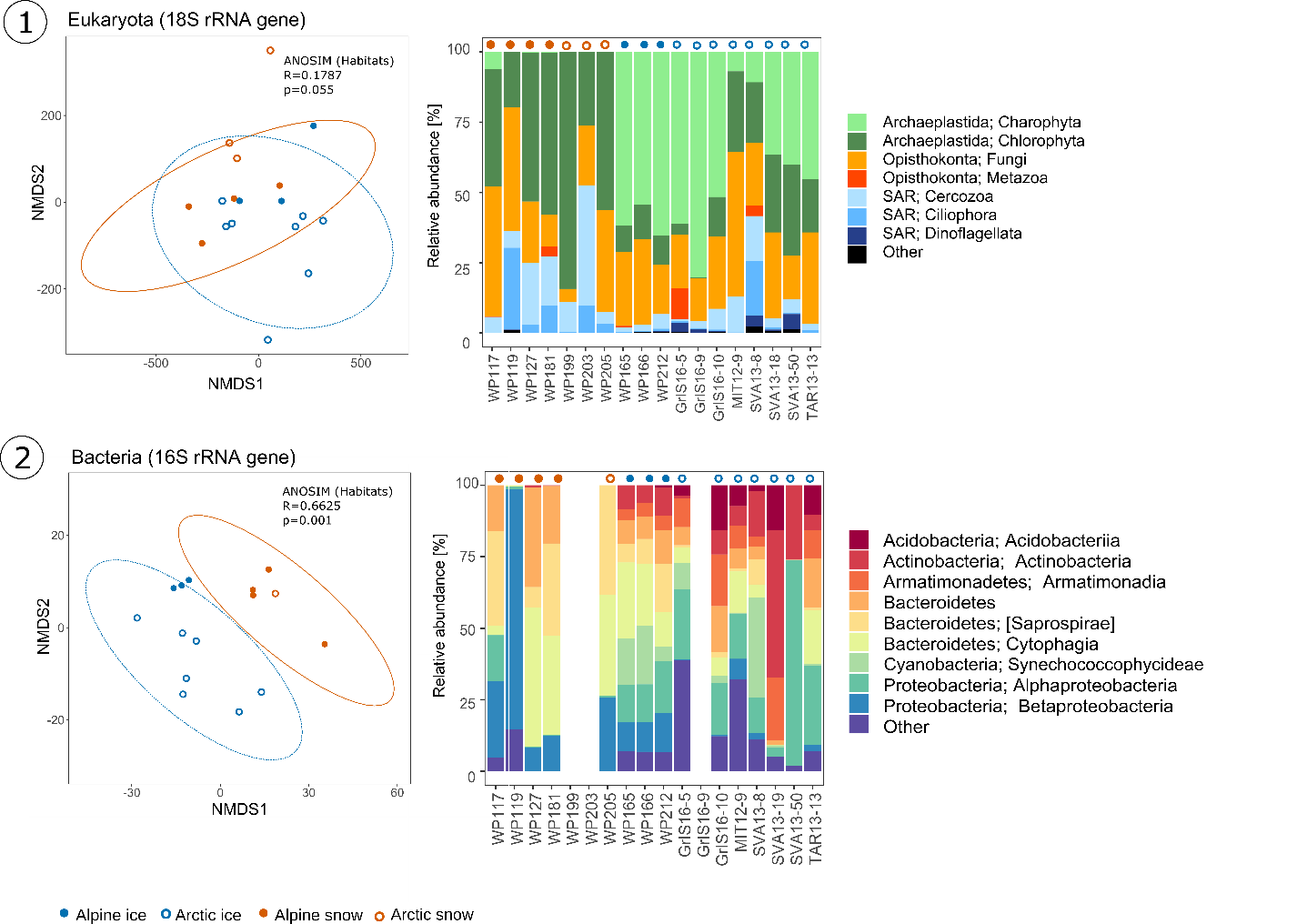
**

**Supplementary Figure 14.** The total bacterial community composition and similarities of snow and glacier surface communities based on amplicon metagenomics of the 16S rRNA markers. The samples were assigned to one of four habitat classes and accordingly labelled in the plot and above the bars: “alpine snow” (orange circle), “arctic snow” (orange ring), “alpine ice” (blue circle) and “arctic ice” (blue ring).

**Supplementary References**

Cepák V, Kvíderová J, Lukavský J. The first description of snow algae on Mount Olympus (Greece). *Nova Hedwigia* 2016;*103(3-4)*:457–473.

Goff LJ, Moon DA, Coleman AW. Molecular delineation of species and species relationships in the red algal agarophytes *Gracilariopsis* and *Gracilaria*. *J Phycol* 1994;30:521–37.

Mikhailyuk TI, Sluiman HJ, Massalski A, Mudimu O, Demchenko EM, Kondratyuk SY, Friedl T. New streptophyte green algae from terrestrial habitats and an assessment of the genus *Interfilum* (Klebsormidiophyceae, Streptophyta). *J Phycol* 2008;44:1586–1603.

Quesada A, Vincent WF. Cyanobacteria in the cryosphere: snow, ice and extreme cold. In: Whitton BA (ed.). *Ecology of cyanobacteria II*. Dordrecht: Springer, 2012, 387–399.

Segawa T, Yonezawa T, Edwards A et al. Biogeography of cryoconite forming cyanobacteria on polar and Asian glaciers. J Biogeogr 2017;44(12):2849–2861.

Seibel PN, Müller T, Dandekar T, Schultz J, Wolf M. 4SALE — a tool for synchronous RNA sequence and secondary structure alignment and editing. *BMC Bioinf* 2006;7:498.

Seibel PN, Müller T, Dandekar T, Wolf M. Synchronous visual analysis and editing of RNA sequence and secondary structure alignments using 4 SALE. *BMC Res Notes* 2008;1:91.

Trumhová K. Diversity of the *Micrasterias papillifera*/*radiosa* (Desmidiales) species complex. Master thesis, Charles University, Czech Republic, 61 pp. 2016.

Takeuchi N, Fujita K, Nakazawa F *et al.* A snow algal community on the surface and in an ice core of Rikha-Samba Glacier in Western Nepali Himalayas. *Bull Glac Res* 2009;*27*:25–35.

Trumhová, K. (2016): Diversity of the *Micrasterias papillifera*/*radiosa* (Desmidiales) species complex. Master thesis, Charles University, Czech Republic, 61 pp.

Vilgalys R, Hester M. Rapid genetic identification and mapping of enzymatically amplified ribosomal DNA from several *Cryptococcus* species. *J Bacteriol.* 1990;172:4238–46.

White TJ, Bruns T, Lee S, Taylor J. Amplification and direct sequencing of fungal ribosomal RNA genes for phylogenetics. In: Innis MA, Gelfand DH, Sninsky JJ, White TJ (eds.). *PCR Protocols: A Guide to Methods and Applications*. New York, USA: Academic Press Inc, 1990:315–322.
